# Supplementary material for: Characteristics and limitations of national antimicrobial surveillance according to sales and claims data
Source: PLoS One. 2021 May 11;16(5):e0251299. doi: 10.1371/journal.pone.0251299 (PMC8112693; doi:10.1371/journal.pone.0251299)
Supplement: Attachment — Submitted filename: PONE-D-20-21601_R1_reviewer_Reviewer2.pdf [file pone.0251299.s002.pdf]

PLOS ONE

Characterization of the differences in national antimicrobial use surveillance between sales data and claims data in Japan

--Manuscript Draft--

**Manuscript Number:**	PONE-D-20-21601R1
**Article Type:**	Research Article
**Full Title:**	Characterization of the differences in national antimicrobial use surveillance between sales data and claims data in Japan
**Short Title:**	Differences in national antimicrobial use between sales and claims data
**Corresponding Author:**	Yoshiki Kusama<br>National Center for Global Health and Medicine Hospital<br>Tokyo, JAPAN
**Keywords:**	antimicrobial use; Antimicrobial resistance; sales data; claim data; surveillance
**Abstract:**	Purpose<br>Countries require rigorous antimicrobial use (AMU) surveillance systems to monitor prescription trends and evaluate the effects of antimicrobial resistance countermeasures. AMU can be estimated using sales data (S-AMU) or insurance claims data (C-AMU), but their differences are not well characterized. We comparatively examined Japan's national S-AMU and C-AMU estimates across a 5-year period, and explored the potential causes of their differences.<br><br>Methods<br>Using wholesalers' drug sales data and national insurance claims data, we calculated the annual S-AMU and C-AMU throughout Japan from 2013 to 2017. AMU was calculated as defined daily doses/1,000 inhabitants/day (DID). We compared annual S-AMU and C-AMU estimates, and evaluated their longitudinal consistency over the study period. In addition, we examined S-AMU and C-AMU according to branded/generic and parenteral/oral classifications, and estimated their correlations using Pearson's correlation coefficient.<br><br>Results<br>The estimated DID values for S-AMU and C-AMU were 14.91 and 14.27 (2013), 14.48 and 14.34 (2014), 14.68 and 15.04 (2015), 14.60 and 14.93 (2016), and 13.81 and 14.12 (2017), respectively. The differences in DID values between S-AMU and C-AMU were 0.65 (2013), 0.12 (2014), -0.37 (2015), -0.32 (2016), and -0.31 (2017). S-AMU and C-AMU were significantly correlated in both branded and generic antimicrobials. Among parenteral drugs, S-AMU was consistently higher than C-AMU for both branded and generic drugs. Among oral drugs, C-AMU was higher than S-AMU for generic drugs, but not branded drugs.<br><br>Conclusions<br>Our study provides insight into the characteristics of S-AMU and C-AMU surveillance in Japan. Their differences may have been influenced by the omission of direct sales information in S-AMU and the lack of electronically submitted claims for dental care in C-AMU. However, these factors did not appear to differentially affect the temporal trends. The differences stabilized after 2015, suggesting that S-AMU is a viable surrogate indicator of Japan's national AMU.
**Order of Authors:**	Yoshiki Kusama
	Yuichi Muraki
	Chika Tanaka
	Ryuji Koizumi
	Masahiro Ishikane
	Daisuke Yamasaki

Powered by Editorial Manager® and ProduXion Manager® from Aries Systems Corporation

	Masaki Tanabe
	Norio Ohmagari
**Response to Reviewers:**	Response to Reviewers<br><br>We have made major revisions to the entire manuscript based on the reviewers' advice and suggestions. We are grateful for the opportunity to improve our work, and look forward to the reviewers' evaluation of the new structure and theories included in the manuscript. Our point-by-point responses to the comments are provided below:<br><br>Reviewer 1<br>This is a very interesting exploration of differences between two comprehensive, population-based methods to observe antibiotic consumption in Japan. The authors are to be commended for performing such analyses in order to understand the available data better and in order to be able to deal with limitations. It is an example for researchers in other nations to do likewise.<br><br>Thank you for your time and effort in reviewing our work, and for your positive evaluation of our manuscript. We have made major revisions in accordance with your comments and suggestions.<br><br>1.The differences found are all by all minimal, which could incite the authors to conclude that Japan has 2 pretty reliable sources to monitor antibiotic consumption. The article should clearly state that this is true for consumption in primary care (and not hospital care). It this would be incorrect it should be stated, and it should also be indicated why it is not possible to make the distinction between primary care and hospital care dispensing and reimbursement.<br><br>Thank you for the advice. S-AMU and C-AMU include both primary care data and hospital data. However, S-AMU does not contain information about where an antibiotic is used, and therefore does not allow us to distinguish between primary care and hospital care dispensing/reimbursement. As advised, we have added the following information to the Data sources subsection of the Methods.<br><br>Page 7 Lines 106-109<br>However, S-AMU data do not include information on the purchasing parties (i.e., names and types of medical facilities) or patients, and therefore do not allow distinctions to be made between primary care and hospital care dispensing and reimbursement.<br><br>2.Some differences were observed but not statistically evaluated. Some explanation why this did not happen might be added (too small differences, complex reversing time lines).<br><br>As advised, we have added more statistical evaluations to our manuscript after consulting with a statistician.<br><br>Page 14 Lines 206-212<br>Effects on parenteral AMU<br>For parenteral antimicrobials, both branded and generic drugs exhibited similar trends in S-AMU and C-AMU (Fig 2A). The use of generic drugs increased while the use of branded drugs decreased over the study period. Furthermore, the scatter plots of branded and generic drugs demonstrated significant correlations between S-AMU ($r = .100$, $P < .001$) and C-AMU ($r = .998$, $P < .001$) (Fig 2B). For both branded and generic drugs, S-AMU was higher than C-AMU throughout the study period.<br><br>Page 15 Lines 223-232<br>Effects on oral AMU<br>The DID values of oral antimicrobials were more than 10 times higher than those of parenteral antimicrobials in both S-AMU and C-AMU. As with the parenteral antimicrobials, both branded and generic drugs in oral antimicrobials exhibited similar trends in S-AMU and C-AMU (Fig 3A). Again, the use of generic drugs increased while the use of branded drugs decreased over the study period. Throughout this period, S-AMU was higher than C-AMU for branded drugs, but C-AMU was higher than S-AMU

Powered by Editorial Manager® and ProduXion Manager® from Aries Systems Corporation

for generic drugs. The scatter plots of branded and generic drugs demonstrated significant correlations between S-AMU ($r = .996$, $P < .001$,) and C-AMU ($r = .997$, $P < .001$) (Fig 3B).

3.The exploration of the small differences leads to hypotheses on potential sources of bias. Most of these hypotheses are plausible. For OTC sales of antibiotics in Japan, the authors states that it is illegal. That does not mean that it does not happens, even in Japan. Some indication about the actual practices in Japan, and some references to the international literature on this should be advisable. It seems plausible that this might have an influence on the observed difference in sales and claims of cheap oral antibiotics (where the remark on the legal status of OTC antibiotics was placed).

Thank you for suggesting this important point. OTC drugs are strictly regulated in Japan, and OTC antibiotics are, for all intents and purposes, not available (excluding some topical preparations). We would be hard pressed to think of any situation where a pharmacy would risk losing their retailing license and face criminal charges for illegally selling antibiotics that are easily and cheaply available with a physician's prescription. In addition, physician consultations/prescriptions are highly affordable under Japan's universal healthcare system. We have searched the international literature on the plausibility of OTC AMU in Japan, but could not find any relevant papers. In addition to the strict legislation on the sale of antibiotics, we think the main reason for this being such a non-issue is simply that the barriers to legally obtaining antibiotics are too low for illegal sales to be worth pursuing.
Although a very small number of personal trades/sales of antimicrobials through websites may occur, such AMU would not be reflected in sales or claims data. We have added these explanations to the limitations as follows:

Page 20 Lines 317-324
First, the analysis provides insight into S-AMU and C-AMU in Japan, but the characteristics and limitations of these data sources may not be directly applicable to those of other countries. For example, other countries may have a non-negligible proportion of antimicrobial sales without prescriptions [23, 24], which can lead to underestimations of C-AMU. In contrast, the inclusion of over-the-counter antimicrobial sales is not an issue in Japan due to strict regulations and easy access to prescribed antimicrobials. In this way, our findings should be evaluated in the context of Japan's healthcare system and AMU patterns.

We have also cited the following additional references.

23.Batista AD, A Rodrigues D, Figueiras A, Zapata-Cachafeiro M, Roque F, Herdeiro MT. Antibiotic Dispensation without a Prescription Worldwide: A Systematic Review. Antibiotics (Basel). 2020;9: E786.
24.Sakeena MHF, Bennett AA, McLachlan AJ. Non-prescription sales of antimicrobial agents at community pharmacies in developing countries: a systematic review. Int J Antimicrob Agents. 2018;52: 771–82.
Reviewer 2
The topic of the paper is of utmost importance when it comes to monitoring AMR. However, I'm under the impression that the work has been hastily prepared. The paper in my view lacks clarity and the methods does not appear sound, unless the authors are dealing with data that lack granularity. In general, thru my read, I'm left wanting more. The sources of my disappointment are as follows:

Thank you for your time and effort in reviewing our work, and we appreciate your advice and comments. The manuscript has undergone a major restructuring.

1.Lack of description of the types of variables or information that are captured by S-AMU and C-AMU

Thank you for your useful suggestion. For greater clarity, we have added explanations of S-AMU and C-AMU as follows:

S-AMU
Page 7, Lines 96-109
We estimated S-AMU using commercial data purchased from IQVIA Japan, a private

data firm that curates databases of medical drug sales and distribution throughout the country. In Japan, medical drugs are generally sold by pharmaceutical companies to medical facilities via wholesalers. IQVIA Japan collects sales data from these wholesalers in order to construct databases. Although detailed information has not been published, the company states that it collects data from almost all domestic wholesalers, and that the data encompass more than 99% (monetary value) of all wholesale drug sales in Japan (this coverage was confirmed through personal communication with IQVIA Japan). Our dataset contained wholesalers' sales data of all antimicrobials, as well as information on oral/parenteral classification and branded/generic classification. However, S-AMU data do not include information on the purchasing parties (i.e., names and types of medical facilities) or patients, and therefore do not allow distinctions to be made between primary care and hospital care dispensing and reimbursement.

C-AMU
Page 8, Lines 112-142

We estimated C-AMU using data obtained from the National Database of Health Insurance Claims and Specific Health Checkups of Japan (NDB), which is managed by the Ministry of Health, Labour and Welfare (MHLW). The NDB contains national-level health insurance claims data for healthcare encounters covered by insurance, and these data can be used for research purposes following the submission and approval of an application to the government [12]. Our dataset included data on the dates and types of antimicrobials prescribed to patients for all insurance-covered healthcare encounters.
In Japan, all residents are required to enroll in health insurance, which entitles them to receive insurance-covered healthcare at any medical facility throughout the country. Enrollees pay monthly premiums to their insurers, and also pay a fixed proportion (10–30% depending on age and income) of the medical charges at the point of care. Healthcare providers send claims to the applicable insurers to be reimbursed for the remaining charges. Because insurance-covered care accounts for the majority of medical treatments provided in Japan, the NDB represents a near-comprehensive database of all treatments performed throughout the country.
However, the NDB does not include claims data from patients with fully publicly funded healthcare (e.g., patients with intractable diseases, atomic bomb survivors, patients on welfare, patients with tuberculosis, and patients with human immunodeficiency virus infections) and patients who personally pay for all of their medical expenses (e.g., foreign travelers and cosmetic surgery patients). The detailed numbers of these patients are not published by the government.
Furthermore, the NDB only includes data from claims that have been submitted electronically, and the lack of paper-based claims reduces its coverage of healthcare encounters. Under Japan's healthcare system, claims for medical care, dental care, and drug dispensing (by pharmacies) are handled separately. From 2011, electronically submitted claims for hospital-based medical care and drug dispensing claims have accounted for over 99.9% of total claims [13]. For clinic-based medical care, the proportions of electronically submitted claims increased from 91.0% in 2011 to 99.9% in 2017 [13]. For dental care, the proportions of electronically submitted claims have increased dramatically in recent years (31.5%, 46.4%, 55.7%, 69.5%, and 96.0% in 2011, 2012, 2013, 2014, and 2015, respectively) [13].

2.Methods look at first glance proper including potential time-series correlations but with limited granular data and its understanding, the authors are left with lots of hypotheses with some sense of triangulation of information/data

We agreed with your opinion, and have revised our study's conceptual framework and development. Previously, we had, as you pointed out, aimed to explore the biases of S-AMU and C-AMU from various aspects. But we have changed this construction: We now consider the effects of the rapid shift in paper-based claims to electronic claims in dental care as a characteristic of C-AMU, and the effects of insufficient data coverage as a characteristic of S-AMU (which is not expected to greatly affect the representativeness of the AMU situation). This simplified the study, and allowed us to focus on the correlation between S-AMU and C-AMU. The specific changes are as follows:

Powered by Editorial Manager® and ProduXion Manager® from Aries Systems Corporation

	Page 14, Lines 209-211<br>Furthermore, the scatter plots of branded and generic drugs demonstrated significant correlations between S-AMU (r = .100, P < .001) and C-AMU (r = .998, P < .001).<br><br>Page 15, Lines 230-232<br>The scatter plots of branded and generic drugs demonstrated significant correlations between S-AMU (r = .996, P < .001,) and C-AMU (r = .997, P < .001).<br><br>3.Emphasis of the authors for the accuracy of AMU data while I believe estimating consistency of the data is much more important: I believe that the verification or validation of the consistency of the reporting from one year to another is more essential. Please recall we mean to use AMU trends as trends to be compared with AMR trends.<br><br>Thank you for the important suggestion. As advised, we have examined the longitudinal consistency of S-AMU compared to C-AMU. The specific changes are as follows:<br><br>Page 10, Lines 159-162<br>To evaluate the longitudinal consistency of S-AMU, we compared the annual S-AMU with C-AMU between 2013 and 2017. In addition, we compared the use of branded and generic drugs in S-AMU and C-AMU to ascertain the effect of the government's initiative to promote the shift to generic drugs.<br><br>4.Indeed, comparison between C-AMU and S-AMU is interesting (if you have access to data, which is not the case here). Both systems could be used to monitor AMR trends as opposed to disqualifying C-AMU but is not capturing comprehensively the data.<br><br>Thank you for the suggestion. We have examined the utility of S-AMU through a comparison with C-AMU in the revised manuscript. The specific changes are as follows:<br><br>Page 18, Lines 275-276<br>These findings indicate that S-AMU has utility as an alternative to C-AMU for monitoring the temporal trends in national AMU.<br><br>5.Perceived some confusion re definitions between accuracy versus precision and between accuracy and biases; e.g. unclear meaning of inadequate accuracy? What does incomplete coverage mean? and moreover incomplete coverage does not mean necessary biases. E.g. incomplete data can be precise if they are representative of a situation.<br><br>Thank you for the comment. As mentioned in our response to Comment 3, we have focused the study on comparing S-AMU and C-AMU. In the revised manuscript, we consider the effects of the rapid shift in paper-based claims to electronic claims in dental care as a characteristic of C-AMU, and the effects of insufficient data coverage as a characteristic of S-AMU.<br><br>Specific comments:<br>6.Line 26: abstract will benefit from a reference to the reason for AMU surveillance: AMR surveillance i.e. why these AMU estimates are important?<br><br>We have clarified the importance of developing rigorous AMU surveillance systems as follows.<br><br>Page 3, Lines 26-32<br>Purpose<br>Countries require rigorous antimicrobial use (AMU) surveillance systems to monitor prescription trends and evaluate the effects of antimicrobial resistance countermeasures. AMU can be estimated using sales data (S-AMU) or insurance claims data (C-AMU), but their differences are not well characterized. We comparatively examined Japan's national S-AMU and C-AMU estimates across a 5-year period, and explored the potential causes of their differences.

	7.Line 34: unclear - as written - why the lack of paper-based claims is a limitation<br><br>Thank you for the comment. The lack of paper-based claims potentially reduces the coverage of C-AMU. However, this may only have an effect on dental care (as reimbursements in the other types of care are predominantly managed by electronic claims). We have clarified this in the Data sources subsection of the Methods.<br><br>8.Line 36: the authors could explain why analyzing direct sales to medical facilities is important; and why medical facilities will only buy generics – how about private hospitals?<br><br>As we have set one of the study's aims to clarify the effect of a government policy that encouraged the transition from branded drugs to generic drugs, the discussion about direct sales has become more important when compared to the previous manuscript. The description has been revised in the Discussion. In Japan, the government determines the drug prices for all administered/prescribed drugs under the national health insurance system. Therefore, patients are charged the same price for a specific drug regardless of where the drug was prescribed (public or private institutes).<br><br>9.Line 37: unclear about the importance of comparison between generic and brand drugs?<br><br>We added the importance of comparing the trends between branded and generic drugs. This was to clarify the effect of a government policy that encouraged the transition from branded drugs to generic drugs.<br><br>Page 6, Lines 82-85<br>In addition, longitudinal consistency is a crucial aspect of a national AMU surveillance system, but antimicrobial sales may be affected by changes to healthcare policies. For example, the Japanese government has encouraged the shift from branded drugs to generic drugs from 2013 onward [10, 11].<br><br>10.Line 38: unclear significance of paper-based and electronic claims. In short, without any understanding on how it works in Japan, I'm having a hard time processing the rationality of the methods<br><br>We apologize for the inadequate explanation. We have addressed the significance of paper-based and electronic claims in the additional explanation of C-AMU, as described in our response to Comment 1.<br><br>11.Line 41 – 44: the authors share DID values and trends. How do we know that these values are statistically significantly different between each other?<br><br>Because S-AMU and C-AMU are created from different data sources, it is difficult to interpret any statistical difference. Instead, we evaluated the correlation between S-AMU and C-AMU. Please refer to our response to Comment 2.<br><br>12.Line 46: interesting that DID of brand drugs are higher than generic drugs. Reasons are not explained, and values are not shown<br><br>The DID values of each group (parenteral/oral, branded/generic, and S-AMU/C-AMU) are shown in Table 1. The higher DID for branded drugs may be due to pharmaceutical companies promoting their sale over generic drugs to healthcare providers, or a large proportion of patients and doctors who choose branded drugs based on perceptions of higher effectiveness and safety. We have added a description of the government's promotion of the transition from branded to generic drugs.<br><br>Page 6, Lines 84-85<br>For example, the Japanese government has encouraged the shift from branded drugs to generic drugs from 2013 onward [10, 11].<br><br>Page 18, Line 277-280<br>The initially high DID for branded drugs may have been due to their promotion by pharmaceutical companies and/or large proportions of patients and physicians who

preferentially chose branded drugs based on perceptions of higher effectiveness and safety.

13.Line 47: what is a dispensing claim

A dispensing claim is an insurance claim for drug dispensing by pharmacies. We have clarified the language as follows.

Page 9, Lines 135-138
Under Japan's healthcare system, claims for medical care, dental care, and drug dispensing (by pharmacies) are handled separately. From 2011, electronically submitted claims for hospital-based medical care and drug dispensing claims have accounted for over 99.9% of total claims [13].

14.Line 51: unclear about the relationship between "AMU is underestimated" and "lack of direct sales"; do the authors mean the data are missing or not analyzable/accessible?

The sentence has been removed from the revised manuscript.

15.Line 51: Conclusion of abstract is weak: lots of results displayed but no indication in the conclusion to make sense of them. E.g. last sentence is a kind of catchall phrase

We have revised the conclusion. It is difficult to meaningfully explain the results for branded/generic drugs and parenteral/oral drugs given the strict word limits of the abstract. Therefore, we have focused on the differences between S-AMU and C-AMU, and concluded that S-AMU may have utility in national AMU surveillance in Japan.

Page 4, Lines 51-56
Our study provides insight into the characteristics of S-AMU and C-AMU surveillance in Japan. Their differences may have been influenced by the omission of direct sales information in S-AMU and the lack of electronically submitted claims for dental care in C-AMU. However, these factors did not appear to differentially affect the temporal trends. The differences stabilized after 2015, suggesting that S-AMU is a viable surrogate indicator of Japan's national AMU.

16.Line 73: the authors argue that previous data did not account for time-series correlations. However, it appears the present study does not show any time-series correlation analysis either, does it?

We have added an analysis of the correlation of S-AMU and C-AMU according to year. Please refer to our response to Comment 2.

17.Line 84: this paragraph on data sources should be expanded to insert much more information on Japan's healthcare systems, detailed description of what is available in terms of variables and time periods of C-AMU and S-AMU. E.g. Private vs public hospitals; vet antibiotics versus antibiotics for humans; only one private company supplying Japan?

We have added more detailed explanations of S-AMU and C-AMU in our response to Comment 1. Furthermore, as advised, we have addressed veterinary AMU in the Discussion with newly cited reports.

Page 20, Lines 310-314
It is also important to adopt a "One Health" approach to controlling antimicrobial resistance, which includes monitoring AMU not only in humans, but also in animals [19]. The Japanese government's "Nippon AMR One Health Report" described the use of S-AMU to calculate the aggregate AMU in both humans and animals [20].

18.Line 105: meaning of inadequate accuracy? Is it important that the data is inaccurate if on can demonstrate that the data is precise and more importantly consistent overtime? If the authors claim inaccuracy – then what is the extent of the inaccuracy?

Powered by Editorial Manager® and ProduXion Manager® from Aries Systems Corporation

We have made major revisions to the manuscript, and the sentences have been deleted.

19.Line 106: just so that I’m clear: S-AMU data pertain to 99% of total sales but direct sales data to medical facilities are missing or not broken down or categorized as such in the dataset?

We apologize for the inadequate explanation. S-AMU data included 99% of wholesale data.

20.Line 117: would be interesting to mention how different these data are different from that of Europe in terms of available variables

We searched for lists of available variables of S-AMU in European countries, and contacted IQVIA about these variables. Unfortunately, we could not obtain any lists that could support such a comparison.

21.Line 120: what is the extent of the AMU among tourists and for cosmetic procedures? Does it matter if we consider the inaccuracy is consistent over time? Recall that trends matter more for AMU and AMR surveillance purposes

We agree with your comments. We have included a study aim to examine the longitudinal consistency between S-AMU and C-AMU, and addressed this lack of data from C-AMU in the limitations.

Page 20, Lines 324-327
Second, information on publicly funded and self-pay patients are not available in the C-AMU. However, this may not greatly affect the longitudinal trends in C-AMU as the number of such patients is likely to be consistent over time.

22.Line 127: what kind of data is shared by IQVIA? Are there any limitations in relation to data sharing? Please detail what is shared by the company and what is available?

We have added a more detailed explanation of the IQVIA data. The purchased data only contained sales of drugs from wholesalers to buyers (mainly medical facilities). IQVIA has restrictions prohibiting the public sharing of the data. However, researchers are free to purchase similar data directly from IQVIA.

23.Line 153: again comparison between the 2 AMU data could be pertinent if these DID values are statistically significant or not?

Thank you for your advice. We have examined the correlations between the 2 AMU data types as detailed in our response to Comment 2.

24.Line 169: IQVIA accounts for 99% of the sales – is this for antimicrobials or all drugs? if data is made available, wouldn’t it be interesting to compare sales of antibiotics with that of other drugs, trends wise? Do you mean IQVIA has the monopoly of sales?

IQVIA accounts for 99% of all wholesale drug sales. Unfortunately, we had only purchased data regarding antimicrobials. While other vendors are free to collect and offer similar data, there are currently none that do so.

25.Line 170: Cant we work out some collaboration with the company for public health purposes to have access to more granular data?

Thank you for your suggestion. IQVIA is unable to provide more granular data due to confidentiality agreements with the wholesalers.

26.Line 175-176: the main result in my view is the consistency of differences over many of these factors (parental vs per os, generic/branded). Shouldn’t the authors emphasize this more in the results?

	Thank you for your suggestions. We have revised our study framework, and emphasize the consistency of the differences between generic vs branded drugs in both oral and parenteral drugs. Please refer to our response to Comment 2.<br><br>27.Line 181 – 182: 2 sentences that say the same thing<br>We have revised the sentence as follows:<br><br>Page 15, Lines 228-230<br>Throughout this period, S-AMU was higher than C-AMU for branded drugs, but C-AMU was higher than S-AMU for generic drugs.<br><br>28.Line 185: figure 2 seems to show interesting trends over time but no analysis?<br><br>We added Pearson's correlation test to these results. Please refer to our response to Comment 2.<br><br>29.Line 198: only one seems to realize that paper-based claims are not submitted into the system.<br><br>We agree with your opinion. We have revised the discussion of paper-based claims such that it is now considered a characteristic of C-AMU.<br><br>30.Line 220 -221: not sure I understand what sort of impact the authors were referring to<br><br>This sentence was deleted as part of the major revisions to the manuscript.<br><br>31.Line 222: difficult to understand this with limited background on how antimicrobials are supplied and distributed<br><br>We have added the following explanation about this statement.<br><br>Page 18, Lines 285-289<br>In general, pharmaceutical companies sell their products to medical facilities through wholesalers to reduce the burden of sales. However, some companies may sell their products directly to medical facilities to increase their profit margins that would otherwise be lost to the wholesalers.<br><br>32.Line 230: the paper becomes much more interesting if there is some attempt to estimate the extent of the biases. Unfortunately, we don't have this in this paper<br><br>Although we agree with this point, we were unable to quantify the extent of these biases with our data. Nevertheless, the study has shifted from speculating about these possible biases, and focused on the differences between C-AMU and S-AMU.<br><br>33.Line 245: I disagree with authors; C-AMU is valuable if consistent overtime<br><br>We have changed the discussion according to your advice, and regarded C-AMU as the standard method for evaluating the national AMU. Please refer to our response to Comment 4.<br><br>34.Line 247: this information could be shared earlier in a potential section on detailed description of AMU data<br><br>Thank you for the comment. However, the need for human-animal aggregate AMU is, although important, not central to our study aims. We have therefore kept this issue in the Discussion.<br><br>35.Line 260: why not doing the analysis of unadjusted DDD in this paper as this paper contains limited information and results<br><br>The reason for using population-adjusted DDD is written in the limitations. Adjusted DID is a more frequently used metric of national AMU than unadjusted DDD, with major international organizations (e.g., WHO and ECDC) using this unit to evaluate the AMU

Powered by Editorial Manager® and ProduXion Manager® from Aries Systems Corporation

	of countries. If readers would like to evaluate unadjusted DDD, they can easily obtain the figures by multiplying DID with the population of Japan, as described in Reference #15 (Statistics of Japan).<br><br>Page 21, Lines 327-332<br>Finally, we evaluated AMU using DDD values adjusted to the national population. Because longitudinal evaluations that use this metric are affected by population changes, unadjusted DDD estimates may represent a more suitable metric to evaluate AMU transitions. Nevertheless, national AMU evaluations frequently use adjusted DID as an indicator, which allows for more practical and intuitive interpretations than unadjusted values.
**Additional Information:**	
**Question**	**Response**
**Financial Disclosure**<br><br>Enter a financial disclosure statement that describes the sources of funding for the work included in this submission. Review the submission guidelines for detailed requirements. View published research articles from *PLOS ONE* for specific examples.<br><br>This statement is required for submission and **will appear in the published article** if the submission is accepted. Please make sure it is accurate.<br><br>**Unfunded studies**<br>Enter: *The author(s) received no specific funding for this work.*<br><br>**Funded studies**<br>Enter a statement with the following details:<br>• Initials of the authors who received each award<br>• Grant numbers awarded to each author<br>• The full name of each funder<br>• URL of each funder website<br>• Did the sponsors or funders play any role in the study design, data collection and analysis, decision to publish, or preparation of the manuscript?<br>• **NO** - Include this sentence at the end of your statement: *The funders had no role in study design, data collection and analysis, decision to publish, or preparation of the manuscript.*<br>• **YES** - Specify the role(s) played.<br><br>* typeset	This study was supported by a research grant from the Ministry of Health, Labour and Welfare of Japan (H29-shinkougyousei-shitei-005).
**Competing Interests**	The authors have declared that no competing interests exist.

Use the instructions below to enter a competing interest statement for this submission. On behalf of all authors, disclose any competing interests that could be perceived to bias this work—acknowledging all financial support and any other relevant financial or non-financial competing interests.<br><br>This statement **will appear in the published article** if the submission is accepted. Please make sure it is accurate. View published research articles from *PLOS ONE* for specific examples.<br><br>**NO authors have competing interests**<br><br>Enter: *The authors have declared that no competing interests exist.*<br><br>**Authors with competing interests**<br><br>Enter competing interest details beginning with this statement:<br><br>*I have read the journal's policy and the authors of this manuscript have the following competing interests: [insert competing interests here]*<br><br>* typeset	
**Ethics Statement**<br><br>Enter an ethics statement for this submission. This statement is required if the study involved:<br><br>• Human participants<br>• Human specimens or tissue<br>• Vertebrate animals or cephalopods<br>• Vertebrate embryos or tissues<br>• Field research<br><br>Write "N/A" if the submission does not require an ethics statement.<br><br>General guidance is provided below. Consult the submission guidelines for detailed instructions. **Make sure that all**	The study did not involve any interventions in human subjects. All IQVIA data and NDB data were fully anonymized before being received by the authors. This study was approved by the institutional review board of the National Center for Global Health and Medicine (Approval Number: NCGM-G-002505-00).

**information entered here is included in the Methods section of the manuscript.**<br><br>**Format for specific study types**<br><br>**Human Subject Research (involving human participants and/or tissue)**<br>• Give the name of the institutional review board or ethics committee that approved the study<br>• Include the approval number and/or a statement indicating approval of this research<br>• Indicate the form of consent obtained (written/oral) or the reason that consent was not obtained (e.g. the data were analyzed anonymously)<br><br>**Animal Research (involving vertebrate animals, embryos or tissues)**<br>• Provide the name of the Institutional Animal Care and Use Committee (IACUC) or other relevant ethics board that reviewed the study protocol, and indicate whether they approved this research or granted a formal waiver of ethical approval<br>• Include an approval number if one was obtained<br>• If the study involved *non-human primates*, add *additional details* about animal welfare and steps taken to ameliorate suffering<br>• If anesthesia, euthanasia, or any kind of animal sacrifice is part of the study, include briefly which substances and/or methods were applied<br><br>**Field Research**<br><br>Include the following details if this study involves the collection of plant, animal, or other materials from a natural setting:<br>• Field permit number<br>• Name of the institution or relevant body that granted permission	
**Data Availability**<br><br>Authors are required to make all data underlying the findings described fully available, without restriction, and from the time of publication. PLOS allows rare exceptions to address legal and ethical	No - some restrictions will apply

Powered by Editorial Manager® and ProduXion Manager® from Aries Systems Corporation

concerns. See the PLOS Data Policy and FAQ for detailed information.<br><br>A Data Availability Statement describing where the data can be found is required at submission. Your answers to this question constitute the Data Availability Statement and **will be published in the article**, if accepted.<br><br>**Important:** Stating 'data available on request from the author' is not sufficient. If your data are only available upon request, select 'No' for the first question and explain your exceptional situation in the text box.<br><br>Do the authors confirm that all data underlying the findings described in their manuscript are fully available without restriction?	
**Describe where the data may be found in full sentences. If you are copying our sample text, replace any instances of XXX with the appropriate details.**<br><br>• If the data are **held or will be held in a public repository**, include URLs, accession numbers or DOIs. If this information will only be available after acceptance, indicate this by ticking the box below. For example: *All XXX files are available from the XXX database (accession number(s) XXX, XXX.*).<br>• If the data are all contained **within the manuscript and/or Supporting Information files**, enter the following: *All relevant data are within the manuscript and its Supporting Information files.*<br>• If neither of these applies but you are able to provide **details of access elsewhere**, with or without limitations, please do so. For example:<br><br>*Data cannot be shared publicly because of [XXX]. Data are available from the XXX Institutional Data Access / Ethics Committee (contact via XXX) for researchers who meet the criteria for access to confidential data.*	Researchers should apply directly to IQVIA Japan (https://www.iqvia.com/ja-jp/locations/japan) for access to the study's antimicrobial drug sales data. The use of the criteria described in the Methods section will enable other researchers to identify the same data obtained for this study. The authors confirm that they did not have any special privileges to access the data that other researchers would not have. For the insurance claims data, the Ministry of Health, Labour and Welfare of Japan has placed strict legal restrictions on the release or sharing of these data. As a result, these data are not publicly available. But researchers can apply for such data as we used to the Ministry of Health, Labour and Welfare of Japan if passed qualification examination (phone number: +81-50-5546-9167).

Powered by Editorial Manager® and ProduXion Manager® from Aries Systems Corporation

The data underlying the results presented in the study are available from (include the name of the third party and contact information or URL).<br>• This text is appropriate if the data are owned by a third party and authors do not have permission to share the data.<br><br>* typeset	
Additional data availability information:	Tick here if the URLs/accession numbers/DOIs will be available only after acceptance of the manuscript for publication so that we can ensure their inclusion before publication.

Powered by Editorial Manager® and ProduXion Manager® from Aries Systems Corporation

Manuscript

Click here to access/download;Manuscript;PONE-D-20-21601_R1_Manuscript_clear_copy.docx

Characterization of the differences in national antimicrobial use surveillance between sales data and claims data in Japan

Yoshiki Kusama[1,2*], Yuichi Muraki[3], Chika Tanaka[1], Ryuji Koizumi[1], Masahiro Ishikane[4], Daisuke Yamasaki[5], Masaki Tanabe[5], Norio Ohmagari[1,2,4]

[1] AMR Clinical Reference Center, Disease Control and Prevention Center, National Center for Global Health and Medicine, Tokyo, Japan

[2] Collaborative Chairs Emerging and Reemerging Infectious Diseases, National Center for Global Health and Medicine, Graduate School of Medicine, Tohoku University, Miyagi, Japan

[3] Department of Clinical Pharmacoepidemiology, Kyoto Pharmaceutical University, Kyoto, Japan

[4] Disease Control and Prevention Center, National Center for Global Health and Medicine, Tokyo, Japan

[5] Department of Infection Control and Prevention, Mie University Hospital, Mie, Japan

*Corresponding author

Email: stone.bagle@gmail.com (YK)

Short running title: Differences in national antimicrobial use between sales and claims data

Abstract (298/300 words)

Purpose

Countries require rigorous antimicrobial use (AMU) surveillance systems to monitor prescription trends and evaluate the effects of antimicrobial resistance countermeasures. AMU can be estimated using sales data (S-AMU) or insurance claims data (C-AMU), but their differences are not well characterized. We comparatively examined Japan’s national S-AMU and C-AMU estimates across a 5-year period, and explored the potential causes of their differences.

Methods

Using wholesalers’ drug sales data and national insurance claims data, we calculated the annual S-AMU and C-AMU throughout Japan from 2013 to 2017. AMU was calculated as defined daily doses/1,000 inhabitants/day (DID). We compared annual S-AMU and C-AMU estimates, and evaluated their longitudinal consistency over the study period. In addition, we examined S-AMU and C-AMU according to branded/generic and parenteral/oral classifications, and estimated their correlations using Pearson’s correlation coefficient.

Results

The estimated DID values for S-AMU and C-AMU were 14.91 and 14.27 (2013), 14.48 and 14.34 (2014), 14.68 and 15.04 (2015), 14.60 and 14.93 (2016), and 13.81 and 14.12 (2017), respectively. The differences in DID values between S-AMU and C-AMU were 0.65 (2013), 0.12 (2014), -0.37 (2015), -0.32 (2016), and -0.31 (2017). S-AMU and C-AMU were significantly correlated in bot[illegible]anded and generic antimicrobials. Among parenteral drugs, S-A[illegible] was consistently higher than C-AMU for both branded and generic drugs. Among oral drugs, C-AMU was higher than S-AMU for generic drugs, but not branded drugs.

Conclusions

[illegible]ur study provides insight into the characteristics of S-AMU and C-AMU surveillance in Japan. Their differences may have been influenced by the omission of direct sales information in S-AMU and the lack of electronically submitted claims for dental care in C-AMU. However, these factors did not appear to differentially affect the temporal trends. The differences stabilized after 2015, suggesting that S[illegible]MU is a viable surrogate indicator of Japan's national AMU.

Introduction

Antimicrobial resistance has rapidly become a global public health and economic issue, and an extensive reduction in antimicrobial use (AMU) is needed to suppress the spread of resistant pathogens [1]. In order to monitor current usage patterns and support the implementation of effective antimicrobial stewardship programs, countries must first establish rigorous national AMU surveillance systems [2].

AMU can be estimated using antimicrobial drug sales data (S-AMU) or insurance claims data (C-AMU). While C-AMU incorporates patient characteristics (e.g., age, sex, and recorded diagnoses), the lack of this information in S-AMU means that the appropriateness of prescriptions cannot be evaluated [3]. Nevertheless, S-AMU may potentially serve as a surrogate indicator of national AMU in countries or situations where C-AMU cannot be easily estimated. A Europe-wide network of national AMU surveillance systems found that 15 countries used S-AMU, 4 countries used C-AMU, and 9 countries used a combination of both in 2018 [4].

In Japan, researchers may acquire national-level health insurance claims data for C-AMU if their applications are approved by the government. However, this process can take a long time as the government prioritizes the protection of patient data. As a result, we have used S-AMU to first publish a preliminary report of annual AMU in

Japan, and subsequently used C-AMU to publish a more comprehensive report [5]. Several studies have also reported on Japan's national AMU trends using sales data [6-8]. However, the differences between S-AMU and C-AMU have not been well characterized in Japan or elsewhere. To the best of our knowledge, only one study has examined the differences between S-AMU and C-AMU [9]. That study found a significant correlation between S-AMU and C-AMU for antimicrobial consumption based on Anatomical Therapeutic Chemical (ATC) classifications, but did not evaluate the cause of the differences. In addition, longitudinal consistency is a crucial aspect of a national AMU surveillance system, but antimicrobial sales may be affected by changes to healthcare policies. For example, the Japanese government has encouraged the shift from branded drugs to generic drugs from 2013 onward [10, 11].

This study aimed to comparatively examine Japan's national S-AMU and C-AMU across a 5-year period, and explore the potential causes of their differences.

Methods

Study design

This retrospective exploratory study was performed to examine the differences between S-AMU and C-AMU, and to evaluate the longitudinal consistency of S-AMU.

Data sources

S-AMU

We estimated S-AMU using commercial data purchased from IQVIA Japan, a private data firm that curates databases of medical drug sales and distribution throughout the country. In Japan, medical drugs are generally sold by pharmaceutical companies to medical facilities via wholesalers. IQVIA Japan collects sales data from these wholesalers in order to construct databases. Although detailed information has not been published, the company states that it collects data from almost all domestic wholesalers, and that the data encompass more than 99% (monetary value) of all wholesale drug sales in Japan (this coverage was confirmed through personal communication with IQVIA Japan). Our dataset contained wholesalers' sales data of all antimicrobials, as well as information on oral/parenteral classification and branded/generic classification. However, S-AMU data do not include information on the purchasing parties (i.e., names and types of medical facilities) or patients, and therefore do not allow distinctions to be made between primary care and hospital care dispensing and reimbursement.

C-AMU

We estimated C-AMU using data obtained from the National Database of Health Insurance Claims and Specific Health Checkups of Japan (NDB), which is managed by the Ministry of Health, Labour and Welfare (MHLW). The NDB contains national-level health insurance claims data for healthcare encounters covered by insurance, and these data can be used for research purposes following the submission and approval of an application to the government [12]. Our dataset included data on the dates and types of antimicrobials prescribed to patients for all insurance-covered healthcare encounters.

In Japan, all residents are required to enroll in health insurance, which entitles them to receive insurance-covered healthcare at any medical facility throughout the country. Enrollees pay monthly premiums to their insurers, and also pay a fixed proportion (10–30% depending on age and income) of the medical charges at the point of care. Healthcare providers send claims to the applicable insurers to be reimbursed for the remaining charges. Because insurance-covered care accounts for the majority of medical treatments provided in Japan, the NDB represents a near-comprehensive database of all treatments performed throughout the country.

However, the NDB does not include claims data from patients with fully publicly funded healthcare (e.g., patients with intractable diseases, atomic bomb

survivors, patients on welfare, patients with tuberculosis, and patients with human immunodeficiency virus infections) and patients who personally pay for all of their medical expenses (e.g., foreign travelers and cosmetic surgery patients). The detailed numbers of these patients are not published by the government.

Furthermore, the NDB only includes data from claims that have been submitted electronically, and the lack of paper-based claims reduces its coverage of healthcare encounters. Under Japan’s healthcare system, claims for medical care, dental care, and drug dispensing (by pharmacies) are handled separately. From 2011, electronically submitted claims for hospital-based medical care and drug dispensing claims have accounted for over 99.9% of total claims [13]. For clinic-based medical care, the proportions of electronically submitted claims increased from 91.0% in 2011 to 99.9% in 2017 [13]. For dental care, the proportions of electronically submitted claims have increased dramatically in recent years (31.5%, 46.4%, 55.7%, 69.5%, and 96.0% in 2011, 2012, 2013, 2014, and 2015, respectively) [13].

AMU measurement

We calculated the annual S-AMU and C-AMU from January 2013 to December 2017. Antimicrobials were identified using the ATC classification of J01, and the

various drug categories were also analyzed according to their relevant ATC codes. AMU was calculated using defined daily dose (DDD) per 1,000 inhabitants per day (DID). This indicator, which shows a drug's assumed average maintenance dose per day, was developed by the World Health Organization Collaborating Centre for Drug Statistics Methodology [14]. DID was calculated using the formula:

$$\mathrm{DID} = \frac{\text{Use in each year (g)}}{\text{DDD (g)} \times \text{population in each year (per inhabitants)} \times \text{365(days)}}$$

Japanese population data were obtained from the MHLW's *Annual Report on Population Dynamics* [15].

Analysis

To evaluate the longitudinal consistency of S-AMU, we compared the annual S-AMU with C-AMU between 2013 and 2017. In addition, we compared the use of branded and generic drugs in S-AMU and C-AMU to ascertain the effect of the government's initiative to promote the shift to generic drugs. We identified drugs as branded or generic based on MHLW's drug classifications [16]. We further evaluated annual S-AMU and C-AMU by the drug administration route (oral or parenteral).

For oral antimicrobials, we evaluated these trends according to high-cost and low-cost antimicrobials. Fluoroquinolones (J01MA) and extended-spectrum penicillins (J01CA) were employed as examples of high-cost and low-cost antimicrobials, respectively. For example, levofloxacin (the most frequently used J01MA drug) costs 355.1 JPY per daily recommended dose, and amoxicillin (the most frequently used J01CA drug) costs 33.3 JPY per daily recommended dose according to the MHLW's drug price list in May 2020 [17]. The correlations between S-AMU and C-AMU according to the branded/generic and parenteral/oral classifications were evaluated using Pearson's correlation coefficient. *P* values below 0.01 were considered statistically significant.

Ethical approval

The study did not involve any interventions in human subjects. All IQVIA data and NDB data were fully anonymized before being received by the authors. This study was approved by the institutional review board of the National Center for Global Health and Medicine (Approval Number: NCGM-G-002505-00).

Results

Trends in total S-AMU and C-AMU from 2013 to 2017

The temporal trends in total S-AMU and C-AMU over the study period are shown in **Fig 1**. The estimated DID values for S-AMU and C-AMU were 14.91 and 14.27 (2013), 14.48 and 14.34 (2014), 14.68 and 15.04 (2015), 14.60 and 14.93 (2016), and 13.81 and 14.12 (2017), respectively. The differences in DID values between S-AMU and C-AMU were 0.65 (2013), 0.12 (2014), -0.37 (2015), -0.32 (2016), and -0.31 (2017). S-AMU produced higher DID estimates than C-AMU in 2013 and 2014, but this gap gradually diminished and was reversed in 2015; thereafter, the differences were relat[illegible]y stable.

Fig 1. National trends in total antimicrobial use in Japan using sales and claims data from 2013 to 2017

C-AMU, antimicrobial use based on claims data; DDD, defined daily doses; S-AMU, antimicrobial use based on sales data.

Effects of the shift from branded to generic drugs on AMU trends

Table 1 presents the trends in DID values of parenteral and oral antimicrobials categorized into branded and generic drugs.

Table 1. Annual antimicrobial use of branded and generic drugs from 2013 to 2017 using sales and claims data

		Data type	2013	2014	2015	2016	2017
Parenteral	Branded	Sales	0.53	0.49	0.46	0.40	0.34
		Claims	0.44	0.41	0.38	0.33	0.28
		Difference	0.09	0.08	0.08	0.07	0.06
	Generic	Sales	0.44	0.47	0.54	0.63	0.71
		Claims	0.39	0.42	0.47	0.53	0.58
		Difference	0.05	0.05	0.07	0.1	0.13
Oral	Branded	Sales	11.67	10.51	9.57	8.39	7.30
		Claims	10.19	9.42	8.82	7.74	6.75
		Difference	1.48	1.09	0.75	0.65	0.55
	Generic	Sales	2.27	3.00	4.10	5.18	5.46

Claims	3.24	4.10	5.37	6.32	6.51
Difference	-0.97	-1.1	-1.27	-1.14	-1.05
Total difference	0.65	0.12	-0.37	-0.32	-0.31

Effects on parenteral AMU

For parenteral antimicrobials, both branded and generic drugs exhibited similar trends in S-AMU and C-AMU (**Fig 2A**). The use of generic drugs increased while the use of branded drugs decreased over the study period. Furthermore, the scatter plots of branded and generic drugs demonstrated significant correlations between S-AMU (r = .100, P < .001) and C-AMU (r = .998, P < .001) (**Fig 2B**). For both branded and generic drugs, S-AMU was higher than C-AMU throughout the study period.

Fig 2. National trends in parenteral antimicrobial use in Japan using sales and claims data for branded and generic drugs from 2013 to 2017

(A) Trends in parenteral antimicrobial use in Japan using sales and claims data for branded and generic drugs from 2013 to 2017

(B) Scatter plots of parenteral antimicrobial use for branded and generic drugs between S-AMU and C-AMU from 2013 to 2017

C-AMU, antimicrobial use based on claims data; DDD, defined daily doses; S-AMU, antimicrobial use based on sales data.

Effects on oral AMU

The DID values of oral antimicrobials were more than 10 times higher than those of parenteral antimicrobials in both S-AMU and C-AMU. As with the parenteral antimicrobials, both branded and generic drugs in oral antimicrobials exhibited similar trends in S-AMU and C-AMU (**Fig 3A**). Again, the use of generic drugs increased while the use of branded drugs decreased over the study period. Throughout this period, S-AMU was higher than C-AMU for branded drugs, but C-AMU was higher than S-AMU for generic drugs. The scatter plots of branded and generic drugs demonstrated significant correlations between S-AMU (r = .996, P < .001,) and C-AMU (r = .997, P < .001) (**Fig 3B**).

Fig 3. National trends in oral antimicrobial use in Japan using sales and claims data for branded and generic drugs from 2013 to 2017

(A) Trends in oral antimicrobial use in Japan using sales and claims data for branded and generic drugs from 2013 to 2017

(B) Scatter plots of oral antimicrobial use for branded and generic drugs between S-AMU and C-AMU from 2013 to 2017

C-AMU, antimicrobial use based on claims data; DDD, defined daily doses; S-AMU, antimicrobial use based on sales data.

Oral AMU for fluoroquinolones and extended-spectrum penicillins

The estimated DID values for S-AMU and C-AMU in fluoroquinolones (high-cost antimicrobials) and extended-spectrum penicillins (low-cost antimicrobials) are shown in **Fig 4**. The range of DID values for fluoroquinolone use between 2013 and 2017 was 2.60–2.87 for S-AMU and 2.74–2.93 for C-AMU. The range of DID values for extended-spectrum penicillin use between 2013 and 2017 was 0.92–1.03 for S-AMU and 0.78–0.95 for C-AMU. S-AMU was consistently higher than C-AMU for extended-spectrum penicillins. In contrast, S-AMU was lower than C-AMU from 2015 to 2017 for fluoroquinolones, which was similar to the trend for total AMU (**Fig 1**).

Fig 4. National trends in oral fluoroquinolone and extended-spectrum penicillin use in Japan using sales and claims data from 2013 to 2017

Oral fluoroquinolones were analyzed as a representative of high-cost antimicrobials, and extended-spectrum penicillins were analyzed as a representative of low-cost antimicrobials. C-AMU, antimicrobial use based on claims data; DDD, defined daily doses; S-AMU, antimicrobial use based on sales data

Discussion

In this retrospective study, we calculated Japan's national S-AMU and C-AMU from 2013 to 2017, and explored the potential causes of their differences. The analysis showed that the DID values differed from 2013 until 2015, but became relatively stable thereafter. These differences may have been influenced by the increased use of electronically submitted claims over the study period and the government's push for a transition from branded to generic drugs.

In 2013 and 2014, the relatively lower use of electronically submitted claims—particularly in dental care—may have led to an underestimation of C-AMU. This manifested as C-AMU having lower national estimates than S-AMU during that period. However, this trend was reversed from 2015 onward, which coincided with the rise in electronically submitted claims. In contrast, the government's push for an increased used of generic drugs from 2013 onward did not appear to substantially affect S-AMU

for both oral and parenteral drugs. While the use of generic drugs consistently increased and the use of branded drugs consistently decreased over the study period, there were no major differences in S-AMU and C-AMU. These findings indicate that S-AMU has utility as an alternative to C-AMU for monitoring the temporal trends in national AMU. The initially high DID for branded drugs may have been due to their promotion by pharmaceutical companies and/or large proportions of patients and physicians who preferentially chose branded drugs based on perceptions of higher effectiveness and safety.

It was interesting to find that C-AMU was higher than S-AMU from 2015 to 2017. S-AMU should be inherently higher than C-AMU because the former includes dead stock and expired medications that are sold to medical facilities but not administered to patients [9]. We posit that this disparity is influenced by the omission of direct sales from pharmaceutical companies to medical facilities in S-AMU. In general, pharmaceutical companies sell their products to medical facilities through wholesalers to reduce the burden of sales. However, some companies may sell their products directly to medical facilities to increase their profit margins that would otherwise be lost to the wholesalers. Our analysis found that S-AMU was only higher than C-AMU for generic oral antimicrobials. Pharmaceutical companies frequently sell generic drugs

directly to medical facilities [18], which may explain this finding. On the other hand, the benefits of direct sales for pharmaceutical companies lie in the exemption of commission charges [18], which are diminished for low-cost drugs. This corroborates our observations that C-AMU was higher than S-AMU for high-cost antimicrobials (fluoroquinolones), and that this pattern was reversed for low-cost antimicrobials (extended-spectrum penicillins). Direct sales to consumers are illegal in Japan, but direct sales to hospitals and clinics are not prohibited. Although we did not find any studies that have addressed these direct sales in other countries, analysts should be aware of their potential effects on national S-AMU surveillance.

Our analysis detected significant correlations between S-AMU and C-AMU for all combinations of parenteral/oral drugs and branded/generic drugs. These findings support the utility of S-AMU (despite the lack of direct sales) in monitoring national AMU trends in Japan. This also supports our strategy to initially publish a preliminary AMU report using S-AMU followed by a more comprehensive report using C-AMU (which includes more detailed information such as stratifications by patient age and sex) [5].

Finally, these findings also underscore a need to characterize and understand the limitations of the various data sources for AMU surveillance in order to accurately

interpret any findings, and that the appropriate surveillance method should be chosen based on the designated purpose. It is also important to adopt a “One Health” approach to controlling antimicrobial resistance, which includes monitoring AMU not only in humans, but also in animals [19]. The Japanese government’s “Nippon AMR One Health Report” described the use of S-AMU to calculate the aggregate AMU in both humans and animals [20]. As sales data are used in drug utilization analyses across various epidemiological fields [21, 22], the findings of this study may be useful not only for researchers of infectious diseases, but also for other conditions.

Our study has several limitations. First, the analysis provides insight into S-AMU and C-AMU in Japan, but the characteristics and limitations of these data sources may not be directly applicable to those of other countries. For example, other countries may have a non-negligible proportion of antimicrobial sales without prescriptions [23, 24], which can lead to underestimations of C-AMU. In contrast, the inclusion of over-the-counter antimicrobial sales is not an issue in Japan due to strict regulations and easy access to prescribed antimicrobials. In this way, our findings should be evaluated in the context of Japan’s healthcare system and AMU patterns. Second, information on publicly funded and self-pay patients are not available in the C-AMU. However, this may not greatly affect the longitudinal trends in C-AMU as the number of such patients

is likely to be consistent over time. Finally, we evaluated AMU using DDD values adjusted to the national population. Because longitudinal evaluations that use this metric are affected by population changes, unadjusted DDD estimates may represent a more suitable metric to evaluate AMU transitions. Nevertheless, national AMU evaluations frequently use adjusted DID as an indicator, which allows for more practical and intuitive interpretations than unadjusted values.

Conclusion

This study comparatively explored the differences in Japan's national AMU estimates from sales and claims data. While S-AMU produced higher DID estimates than C-AMU in 2013 and 2014, this trend reversed and stabilized after 2015. The differences may have been affected by the omission of direct sales information in S-AMU and the lack of electronically submitted claims in C-AMU in the earlier half of the study period. An understanding of each data source's characteristics may facilitate more accurate interpretations of national AMU estimates. These findings provide insight into the differences in Japan's S-AMU and C-AMU surveillance, and indicate that S-AMU is a viable surrogate indicator of Japan's national AMU.

Acknowledgments

None.

References

1. World Health Organization. Surveillance of Antimicrobial Use. [cited 2020 Apr 16]. Available from: http://www.who.int/medicines/areas/rational_use/AMU_Surveillance/en/
2. Pulcini C, Hulscher M. How Can We Routinely Measure Appropriateness of Antimicrobial Use in Hospitals at a National Level? JAMA Netw Open. 2019;2: e1915030. doi: 10.1001/jamanetworkopen.2019.15030
3. Pereko DD, Lubbe MS, Essack SY. Surveillance of antibiotic use in the private sector in Namibia using sales and claims data. J Infect Dev Ctries. 2016;10: 1243–1249
4. Data source overview of antimicrobial consumption. European Centre for Disease Prevention and Control. [cited 2020 Apr 17]. Available from: https://www.ecdc.europa.eu/en/antimicrobial-consumption/database/data-source-overview
5. AMR Clinical Reference Center. Surveillance. [cited 2020 Oct 30]. Available

from: http://amrcrc.ncgm.go.jp/surveillance/Surveillance_en.html

6. Muraki Y, Yagi T, Tsuji Y, Nishimura N, Tanabe M, Niwa T, et al. Japanese antimicrobial consumption surveillance: First report on oral and parenteral antimicrobial consumption in Japan (2009-2013). J Glob Antimicrob Resist. 2016;7:19–23.
7. Tsutsui A, Yahara K, Shibayama K. Trends and patterns of national antimicrobial consumption in Japan from 2004 to 2016. J Infect Chemother. 2018;24:414–21.
8. Kusama Y, Ishikane M, Tanaka C, Kimura Y, Yumura E, Hayakawa K, et al. Regional Variation of Antimicrobial Use in Japan from 2013-2016, as Estimated by the Sales Data. Jpn J Infect Dis. 2019;72:326–9.
9. Yamasaki D, Tanabe M, Muraki Y, Kato G, Ohmagari N, Yagi T. The first report of Japanese antimicrobial use measured by national database based on health insurance claims data (2011–2013): comparison with sales data, and trend analysis stratified by antimicrobial category and age group. Infection. 2018;46: 207–214.
10. Kawabe A, Muraki Y, Inose R, Kusama Y, Goto R, Ebisui A, et al. Trends of Antifungal Use Based on Sales Data in Japan from 2006 to 2015. Biol Pharm Bull. 2020;43: 1248–52.
11. Goto R, Inose R, Kusama Y, Kawabe A, Ishii S, Ebisui A, et al. Trends of the use

of anti-methicillin-resistant Staphylococcus aureus agents in Japan based on sales data from 2006 to 2015. Biol Pharm Bull (in press).

12. Matsuda S, Fujimori K. The claim database in Japan. Asian Pac J Dis Manag. 2012;6: 55–9.
13. Ministry of Health, Labour and Welfare of Japan. Situation in the prevalence of the claim submission via electronic devices (in Japanese). 2015 April [cited 2020 Apr 17]. Available from: https://www.mhlw.go.jp/stf/seisakunitsuite/bunya/0000099015.html
14. WHO Collaborating Centre for Drug Statistics Methodology. ATC/DDD Index 2019. [cited 2020 Nov 18] https://www.whocc.no/atc_ddd_index/
15. The National Statistics Center. Statistics of Japan. [cited 2020 June 1] Available from: https://www.e-stat.go.jp/en
16. Ministry of Health, Labor, and Welfare. Information of drug lists and of generic drugs (in Japanese). 2020 May 27 [cited 2020 June 1] Available from https://www.mhlw.go.jp/topics/2020/04/tp20200401-01.html.
17. Ministry of Health Labour, and Welfare of Japan. National health insurance drug price list and information of generic drugs (in Japanese). 2020 April 1 [cited 2020 Apr 17]. Available from: https://www.mhlw.go.jp/topics/2020/04/tp20200401-

01.html
18. Grüne G, Lockemann S, Kluy V, Meinhardt S. Business Process Management within Chemical and Pharmaceutical Industries. Berlin: Springer-Verlag Berlin Heidelberg; 2014.
19. White A, Hughes JM. Critical Importance of a One Health Approach to Antimicrobial Resistance. EcoHealth. 2019;16:404–9.
20. The AMR One Health Surveillance Committee. Nippon AMR One Health Report (NAOR) 2018. Tokyo: Tuberculosis and Infectious Diseases Control Division, Health Service Bureau, Ministry of Health, Labour and Welfare; 2018
21. Shibata S, Wayama Y, Tsuyuki A, Matsushita M, Chiba K, Matsuki E, et al. An Empirical Study of the Prescription Pattern of Drugs for Hematological Malignancies in Japan from 2010-2014. Biol Pharm Bull. 2017;40: 894–901.
22. Mahlich J, Kamae I, Sruamsiri R. Pharmaceutical pricing in Japan: market evidence for rheumatoid arthritis treatment. Expert Rev Pharmacoecon Outcomes Res. 2018;18: 339–348.
23. Batista AD, A Rodrigues D, Figueiras A, Zapata-Cachafeiro M, Roque F, Herdeiro MT. Antibiotic Dispensation without a Prescription Worldwide: A Systematic Review. Antibiotics (Basel). 2020;9: E786.

24. Sakeena MHF, Bennett AA, McLachlan AJ. Non-prescription sales of antimicrobial agents at community pharmacies in developing countries: a systematic review. Int J Antimicrob Agents. 2018;52: 771–82.

Fig1

Click here to access/download;Figure;PONE-D-20-21601_R1_Fig1.tiff

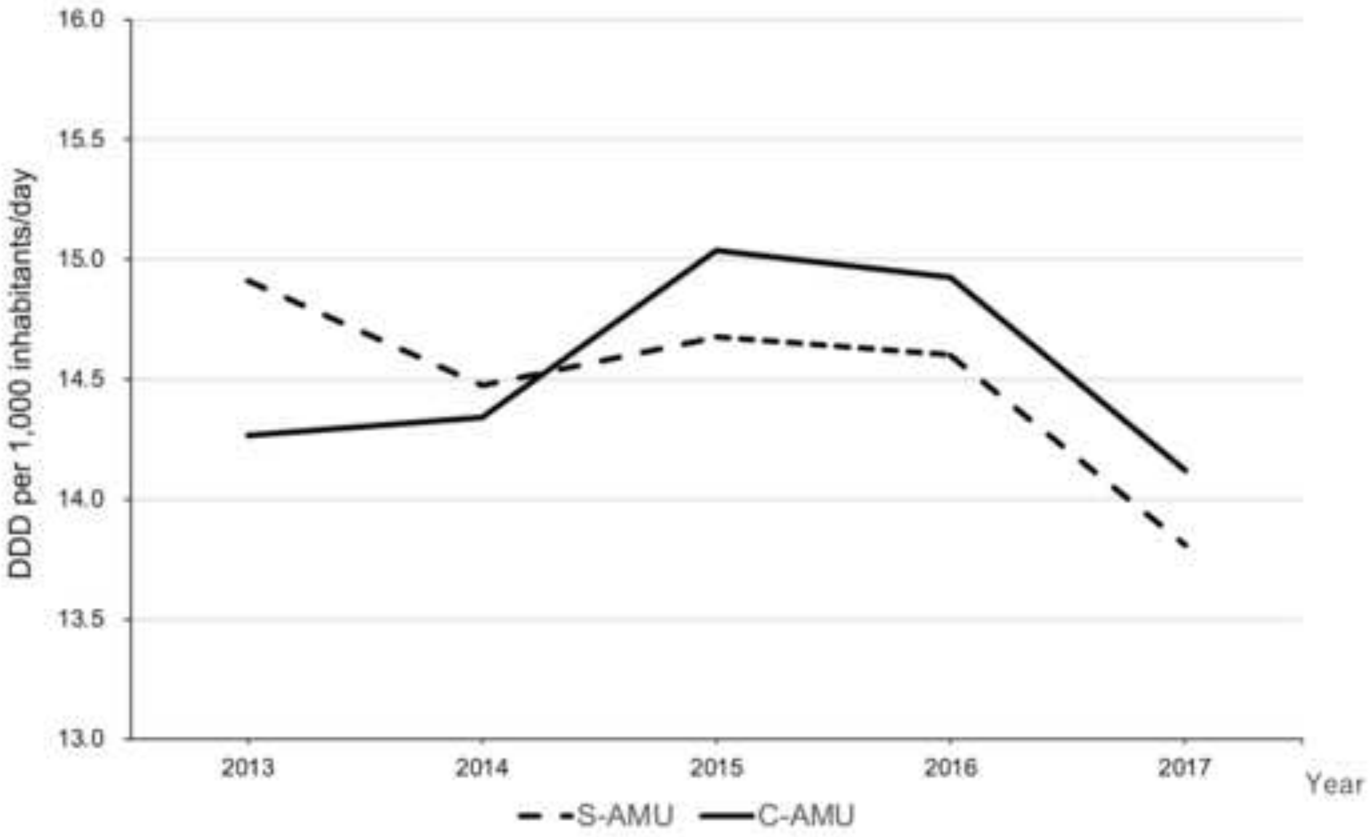

Fig2

Click here to access/download;Figure;PONE-D-20-21601_R1_Fig2.tiff

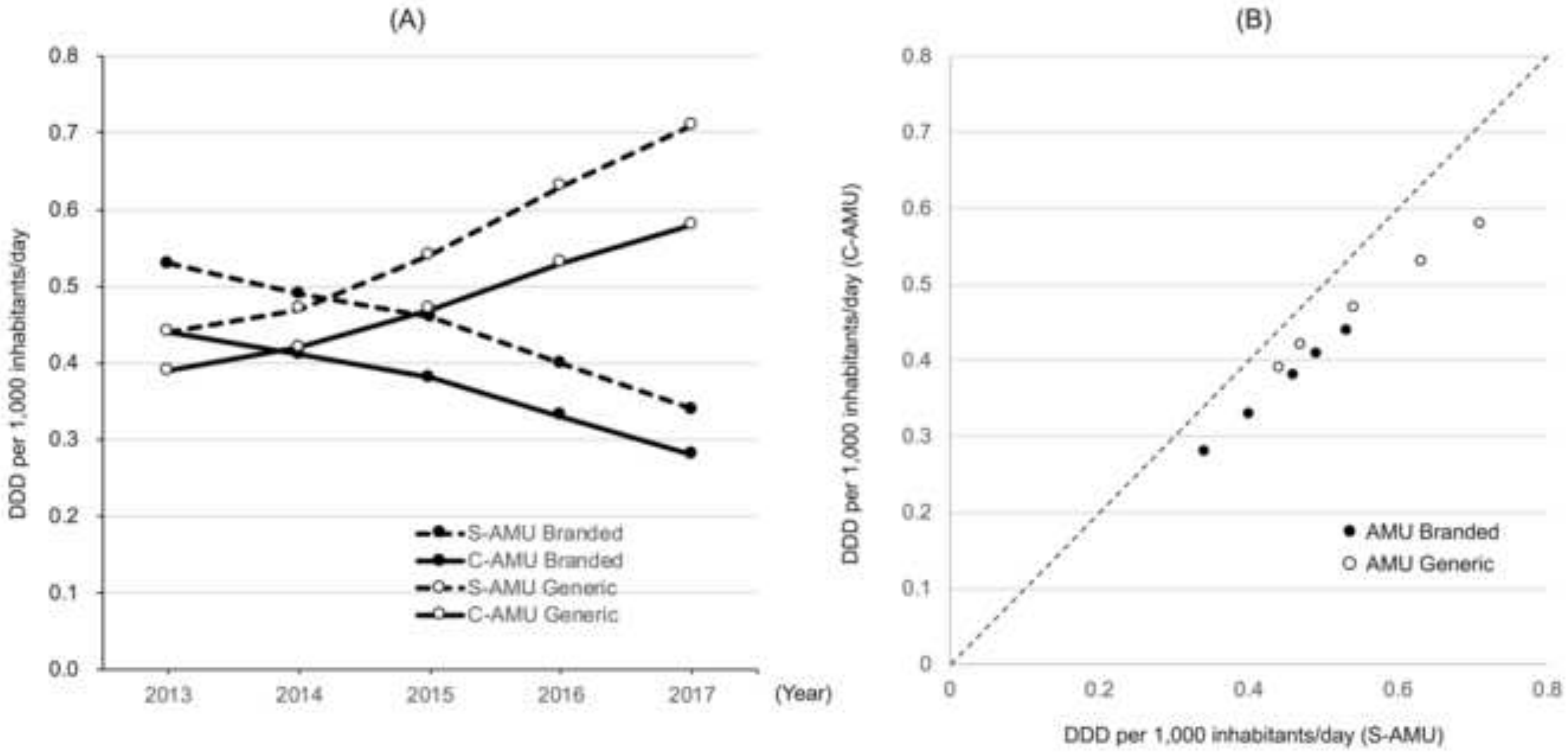

Fig3

Click here to access/download;Figure;PONE-D-20-21601_R1_Fig3.tiff

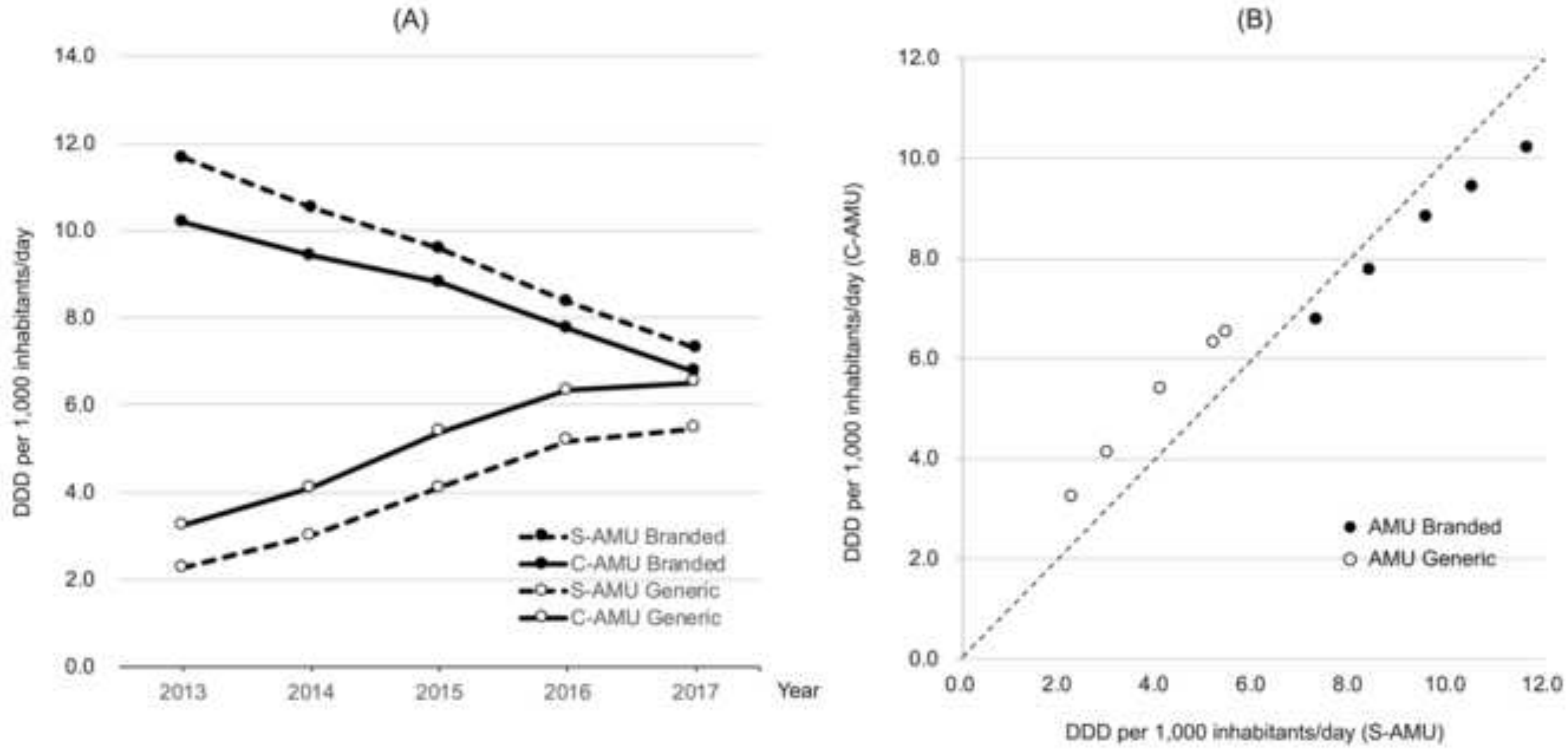

Click here to access/download;Figure;PONE-D-20-21601_R1_Fig4.tiff

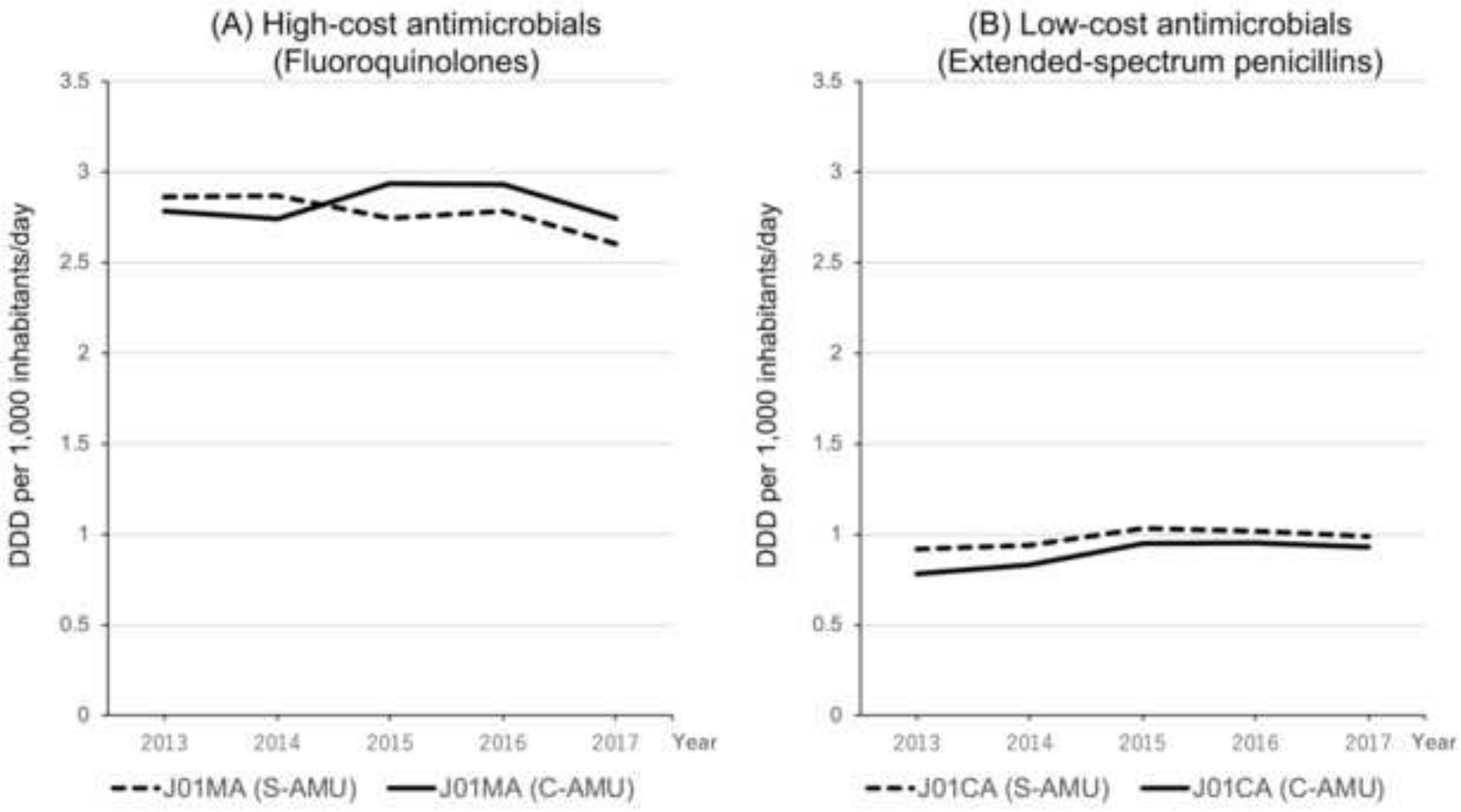

Characterization of the differences in national antimicrobial use surveillance between sales data and claims data in Japan

Yoshiki Kusama[1,2*], Yuichi Muraki[3], Chika Tanaka[1], Ryuji Koizumi[1], Masahiro Ishikane[4], Daisuke Yamasaki[5], Masaki Tanabe[5], Norio Ohmagari[1,2,4]

[1] AMR Clinical Reference Center, Disease Control and Prevention Center, National Center for Global Health and Medicine, Tokyo, Japan

[2] Collaborative Chairs Emerging and Reemerging Infectious Diseases, National Center for Global Health and Medicine, Graduate School of Medicine, Tohoku University, Miyagi, Japan

[3] Department of Clinical Pharmacoepidemiology, Kyoto Pharmaceutical University, Kyoto, Japan

[4] Disease Control and Prevention Center, National Center for Global Health and Medicine, Tokyo, Japan

[5] Department of Infection Control and Prevention, Mie University Hospital, Mie, Japan

*Corresponding author

Email: stone.bagle@gmail.com (YK)

Short running title: Differences in national antimicrobial use between sales and claims data

Abstract (298/300 words)

Purpose

Countries require rigorous antimicrobial use (AMU) surveillance systems to monitor prescription trends and evaluate the effects of antimicrobial resistance countermeasures. AMU can be estimated using sales data (S-AMU) or insurance claims data (C-AMU), but their differences are not well characterized. We comparatively examined Japan's national S-AMU and C-AMU estimates across a 5-year period, and explored the potential causes of their differences.

Methods

Using wholesalers' drug sales data and national insurance claims data, we calculated the annual S-AMU and C-AMU throughout Japan from 2013 to 2017. AMU was calculated as defined daily doses/1,000 inhabitants/day (DID). We compared annual S-AMU and C-AMU estimates, and evaluated their longitudinal consistency over the study period. In addition, we examined S-AMU and C-AMU according to branded/generic and parenteral/oral classifications, and estimated their correlations using Pearson's correlation coefficient.

Results

The estimated DID values for S-AMU and C-AMU were 14.91 and 14.27 (2013), 14.48 and 14.34 (2014), 14.68 and 15.04 (2015), 14.60 and 14.93 (2016), and 13.81 and 14.12 (2017), respectively. The differences in DID values between S-AMU and C-AMU were 0.65 (2013), 0.12 (2014), -0.37 (2015), -0.32 (2016), and -0.31 (2017). S-AMU and C-AMU were significantly correlated in both branded and generic antimicrobials. Among parenteral drugs, S-AMU was consistently higher than C-AMU for both branded and generic drugs. Among oral drugs, C-AMU was higher than S-AMU for generic drugs, but not branded drugs.

Conclusions

Our study provides insight into the characteristics of S-AMU and C-AMU surveillance in Japan. Their differences may have been influenced by the omission of direct sales information in S-AMU and the lack of electronically submitted claims for dental care in C-AMU. However, these factors did not appear to differentially affect the temporal trends. The differences stabilized after 2015, suggesting that S-AMU is a viable surrogate indicator of Japan’s national AMU.

Introduction

Antimicrobial resistance has rapidly become a global public health and economic issue, and an extensive reduction in antimicrobial use (AMU) is needed to suppress the spread of resistant pathogens [1]. In order to monitor current usage patterns and support the implementation of effective antimicrobial stewardship programs, countries must first establish rigorous national AMU surveillance systems [2].

AMU can be estimated using antimicrobial drug sales data (S-AMU) or insurance claims data (C-AMU). While C-AMU incorporates patient characteristics (e.g., age, sex, and recorded diagnoses), the lack of this information in S-AMU means that the appropriateness of prescriptions cannot be evaluated [3]. Nevertheless, S-AMU may potentially serve as a surrogate indicator of national AMU in countries or situations where C-AMU cannot be easily estimated. A Europe-wide network of national AMU surveillance systems found that 15 countries used S-AMU, 4 countries used C-AMU, and 9 countries used a combination of both in 2018 [4].

In Japan, researchers may acquire national-level health insurance claims data for C-AMU if their applications are approved by the government. However, this process can take a long time as the government prioritizes the protection of patient data. As a result, we have used S-AMU to first publish a preliminary report of annual AMU in

Japan, and subsequently used C-AMU to publish a more comprehensive report [5]. Several studies have also reported on Japan's national AMU trends using sales data [6-8]. However, the differences between S-AMU and C-AMU have not been well characterized in Japan or elsewhere. To the best of our knowledge, only one study has examined the differences between S-AMU and C-AMU [9]. That study found a significant correlation between S-AMU and C-AMU for antimicrobial consumption based on Anatomical Therapeutic Chemical (ATC) classifications, but did not evaluate the cause of the differences. In addition, longitudinal consistency is a crucial aspect of a national AMU surveillance system, but antimicrobial sales may be affected by changes to healthcare policies. For example, the Japanese government has encouraged the shift from branded drugs to generic drugs from 2013 onward [10, 11].

This study aimed to comparatively examine Japan's national S-AMU and C-AMU across a 5-year period, and explore the potential causes of their differences.

Methods

Study design

This retrospective exploratory study was performed to examine the differences between S-AMU and C-AMU, and to evaluate the longitudinal consistency of S-AMU.

Data sources

S-AMU

We estimated S-AMU using commercial data purchased from IQVIA Japan, a private data firm that curates databases of medical drug sales and distribution throughout the country. In Japan, medical drugs are generally sold by pharmaceutical companies to medical facilities via wholesalers. IQVIA Japan collects sales data from these wholesalers in order to construct databases. Although detailed information has not been published, the company states that it collects data from almost all domestic wholesalers, and that the data encompass more than 99% (monetary value) of all wholesale drug sales in Japan (this coverage was confirmed through personal communication with IQVIA Japan). Our dataset contained wholesalers' sales data of all antimicrobials, as well as information on oral/parenteral classification and branded/generic classification. However, S-AMU data do not include information on the purchasing parties (i.e., names and types of medical facilities) or patients, and therefore do not allow distinctions to be made between primary care and hospital care dispensing and reimbursement.

C-AMU

We estimated C-AMU using data obtained from the National Database of Health Insurance Claims and Specific Health Checkups of Japan (NDB), which is managed by the Ministry of Health, Labour and Welfare (MHLW). The NDB contains national-level health insurance claims data for healthcare encounters covered by insurance, and these data can be used for research purposes following the submission and approval of an application to the government [12]. Our dataset included data on the dates and types of antimicrobials prescribed to patients for all insurance-covered healthcare encounters.

In Japan, all residents are required to enroll in health insurance, which entitles them to receive insurance-covered healthcare at any medical facility throughout the country. Enrollees pay monthly premiums to their insurers, and also pay a fixed proportion (10–30% depending on age and income) of the medical charges at the point of care. Healthcare providers send claims to the applicable insurers to be reimbursed for the remaining charges. Because insurance-covered care accounts for the majority of medical treatments provided in Japan, the NDB represents a near-comprehensive database of all treatments performed throughout the country.

However, the NDB does not include claims data from patients with fully publicly funded healthcare (e.g., patients with intractable diseases, atomic bomb

survivors, patients on welfare, patients with tuberculosis, and patients with human immunodeficiency virus infections) and patients who personally pay for all of their medical expenses (e.g., foreign travelers and cosmetic surgery patients). The detailed numbers of these patients are not published by the government.

Furthermore, the NDB only includes data from claims that have been submitted electronically, and the lack of paper-based claims reduces its coverage of healthcare encounters. Under Japan’s healthcare system, claims for medical care, dental care, and drug dispensing (by pharmacies) are handled separately. From 2011, electronically submitted claims for hospital-based medical care and drug dispensing claims have accounted for over 99.9% of total claims [13]. For clinic-based medical care, the proportions of electronically submitted claims increased from 91.0% in 2011 to 99.9% in 2017 [13]. For dental care, the proportions of electronically submitted claims have increased dramatically in recent years (31.5%, 46.4%, 55.7%, 69.5%, and 96.0% in 2011, 2012, 2013, 2014, and 2015, respectively) [13].

AMU measurement

We calculated the annual S-AMU and C-AMU from January 2013 to December 2017. Antimicrobials were identified using the ATC classification of J01, and the

various drug categories were also analyzed according to their relevant ATC codes. AMU was calculated using defined daily dose (DDD) per 1,000 inhabitants per day (DID). This indicator, which shows a drug's assumed average maintenance dose per day, was developed by the World Health Organization Collaborating Centre for Drug Statistics Methodology [14]. DID was calculated using the formula:

$$\mathrm{DID} = \frac{\text{Use in each year (g)}}{\text{DDD (g)} \times \text{population in each year (per inhabitants)} \times 365\text{(days)}}$$

Japanese population data were obtained from the MHLW's *Annual Report on Population Dynamics* [15].

Analysis

To evaluate the longitudinal consistency of S-AMU, we compared the annual S-AMU with C-AMU between 2013 and 2017. In addition, we compared the use of branded and generic drugs in S-AMU and C-AMU to ascertain the effect of the government's initiative to promote the shift to generic drugs. We identified drugs as branded or generic based on MHLW's drug classifications [16]. We further evaluated annual S-AMU and C-AMU by the drug administration route (oral or parenteral).

For oral antimicrobials, we evaluated these trends according to high-cost and low-cost antimicrobials. Fluoroquinolones (J01MA) and extended-spectrum penicillins (J01CA) were employed as examples of high-cost and low-cost antimicrobials, respectively. For example, levofloxacin (the most frequently used J01MA drug) costs 355.1 JPY per daily recommended dose, and amoxicillin (the most frequently used J01CA drug) costs 33.3 JPY per daily recommended dose according to the MHLW's drug price list in May 2020 [17]. The correlations between S-AMU and C-AMU according to the branded/generic and parenteral/oral classifications were evaluated using Pearson's correlation coefficient. *P* values below 0.01 were considered statistically significant.

Ethical approval

The study did not involve any interventions in human subjects. All IQVIA data and NDB data were fully anonymized before being received by the authors. This study was approved by the institutional review board of the National Center for Global Health and Medicine (Approval Number: NCGM-G-002505-00).

Results

Trends in total S-AMU and C-AMU from 2013 to 2017

The temporal trends in total S-AMU and C-AMU over the study period are shown in **Fig 1**. The estimated DID values for S-AMU and C-AMU were 14.91 and 14.27 (2013), 14.48 and 14.34 (2014), 14.68 and 15.04 (2015), 14.60 and 14.93 (2016), and 13.81 and 14.12 (2017), respectively. The differences in DID values between S-AMU and C-AMU were 0.65 (2013), 0.12 (2014), -0.37 (2015), -0.32 (2016), and -0.31 (2017). S-AMU produced higher DID estimates than C-AMU in 2013 and 2014, but this gap gradually diminished and was reversed in 2015; thereafter, the differences were relatively stable.

Fig 1. National trends in total antimicrobial use in Japan using sales and claims data from 2013 to 2017

C-AMU, antimicrobial use based on claims data; DDD, defined daily doses; S-AMU, antimicrobial use based on sales data.

Effects of the shift from branded to generic drugs on AMU trends

Table 1 presents the trends in DID values of parenteral and oral antimicrobials categorized into branded and generic drugs.

Table 1. Annual antimicrobial use of branded and generic drugs from 2013 to 2017 using sales and claims data

		Data type	2013	2014	2015	2016	2017
Parenteral	Branded	Sales	0.53	0.49	0.46	0.40	0.34
		Claims	0.44	0.41	0.38	0.33	0.28
		Difference	0.09	0.08	0.08	0.07	0.06
	Generic	Sales	0.44	0.47	0.54	0.63	0.71
		Claims	0.39	0.42	0.47	0.53	0.58
		Difference	0.05	0.05	0.07	0.1	0.13
Oral	Branded	Sales	11.67	10.51	9.57	8.39	7.30
		Claims	10.19	9.42	8.82	7.74	6.75
		Difference	1.48	1.09	0.75	0.65	0.55
	Generic	Sales	2.27	3.00	4.10	5.18	5.46

Claims	3.24	4.10	5.37	6.32	6.51
Difference	-0.97	-1.1	-1.27	-1.14	-1.05
Total difference	0.65	0.12	-0.37	-0.32	-0.31

Effects on parenteral AMU

For parenteral antimicrobials, both branded and generic drugs exhibited similar trends in S-AMU and C-AMU (**Fig 2A**). The use of generic drugs increased while the use of branded drugs decreased over the study period. Furthermore, the scatter plots of branded and generic drugs demonstrated significant correlations between S-AMU (r = .100, P < .001) and C-AMU (r = .998, P < .001) (**Fig 2B**). For both branded and generic drugs, S-AMU was higher than C-AMU throughout the study period.

Fig 2. National trends in parenteral antimicrobial use in Japan using sales and claims data for branded and generic drugs from 2013 to 2017

(A) Trends in parenteral antimicrobial use in Japan using sales and claims data for branded and generic drugs from 2013 to 2017

(B) Scatter plots of parenteral antimicrobial use for branded and generic drugs between S-AMU and C-AMU from 2013 to 2017

C-AMU, antimicrobial use based on claims data; DDD, defined daily doses; S-AMU, antimicrobial use based on sales data.

Effects on oral AMU

The DID values of oral antimicrobials were more than 10 times higher than those of parenteral antimicrobials in both S-AMU and C-AMU. As with the parenteral antimicrobials, both branded and generic drugs in oral antimicrobials exhibited similar trends in S-AMU and C-AMU (**Fig 3A**). Again, the use of generic drugs increased while the use of branded drugs decreased over the study period. Throughout this period, S-AMU was higher than C-AMU for branded drugs, but C-AMU was higher than S-AMU for generic drugs. The scatter plots of branded and generic drugs demonstrated significant correlations between S-AMU (r = .996, P < .001,) and C-AMU (r = .997, P < .001) (**Fig 3B**).

Fig 3. National trends in oral antimicrobial use in Japan using sales and claims data for branded and generic drugs from 2013 to 2017

(A) Trends in oral antimicrobial use in Japan using sales and claims data for branded and generic drugs from 2013 to 2017

(B) Scatter plots of oral antimicrobial use for branded and generic drugs between S-AMU and C-AMU from 2013 to 2017

C-AMU, antimicrobial use based on claims data; DDD, defined daily doses; S-AMU, antimicrobial use based on sales data.

Oral AMU for fluoroquinolones and extended-spectrum penicillins

The estimated DID values for S-AMU and C-AMU in fluoroquinolones (high-cost antimicrobials) and extended-spectrum penicillins (low-cost antimicrobials) are shown in **Fig 4**. The range of DID values for fluoroquinolone use between 2013 and 2017 was 2.60–2.87 for S-AMU and 2.74–2.93 for C-AMU. The range of DID values for extended-spectrum penicillin use between 2013 and 2017 was 0.92–1.03 for S-AMU and 0.78–0.95 for C-AMU. S-AMU was consistently higher than C-AMU for extended-spectrum penicillins. In contrast, S-AMU was lower than C-AMU from 2015 to 2017 for fluoroquinolones, which was similar to the trend for total AMU (**Fig 1**).

Fig 4. National trends in oral fluoroquinolone and extended-spectrum penicillin use in Japan using sales and claims data from 2013 to 2017

Oral fluoroquinolones were analyzed as a representative of high-cost antimicrobials, and extended-spectrum penicillins were analyzed as a representative of low-cost antimicrobials. C-AMU, antimicrobial use based on claims data; DDD, defined daily doses; S-AMU, antimicrobial use based on sales data

Discussion

In this retrospective study, we calculated Japan's national S-AMU and C-AMU from 2013 to 2017, and explored the potential causes of their differences. The analysis showed that the DID values differed from 2013 until 2015, but became relatively stable thereafter. These differences may have been influenced by the increased use of electronically submitted claims over the study period and the government's push for a transition from branded to generic drugs.

In 2013 and 2014, the relatively lower use of electronically submitted claims—particularly in dental care—may have led to an underestimation of C-AMU. This manifested as C-AMU having lower national estimates than S-AMU during that period. However, this trend was reversed from 2015 onward, which coincided with the rise in electronically submitted claims. In contrast, the government's push for an increased used of generic drugs from 2013 onward did not appear to substantially affect S-AMU

for both oral and parenteral drugs. While the use of generic drugs consistently increased and the use of branded drugs consistently decreased over the study period, there were no major differences in S-AMU and C-AMU. These findings indicate that S-AMU has utility as an alternative to C-AMU for monitoring the temporal trends in national AMU. The initially high DID for branded drugs may have been due to their promotion by pharmaceutical companies and/or large proportions of patients and physicians who preferentially chose branded drugs based on perceptions of higher effectiveness and safety.

It was interesting to find that C-AMU was higher than S-AMU from 2015 to 2017. S-AMU should be inherently higher than C-AMU because the former includes dead stock and expired medications that are sold to medical facilities but not administered to patients [9]. We posit that this disparity is influenced by the omission of direct sales from pharmaceutical companies to medical facilities in S-AMU. In general, pharmaceutical companies sell their products to medical facilities through wholesalers to reduce the burden of sales. However, some companies may sell their products directly to medical facilities to increase their profit margins that would otherwise be lost to the wholesalers. Our analysis found that S-AMU was only higher than C-AMU for generic oral antimicrobials. Pharmaceutical companies frequently sell generic drugs

directly to medical facilities [18], which may explain this finding. On the other hand, the benefits of direct sales for pharmaceutical companies lie in the exemption of commission charges [18], which are diminished for low-cost drugs. This corroborates our observations that C-AMU was higher than S-AMU for high-cost antimicrobials (fluoroquinolones), and that this pattern was reversed for low-cost antimicrobials (extended-spectrum penicillins). Direct sales to consumers are illegal in Japan, but direct sales to hospitals and clinics are not prohibited. Although we did not find any studies that have addressed these direct sales in other countries, analysts should be aware of their potential effects on national S-AMU surveillance.

Our analysis detected significant correlations between S-AMU and C-AMU for all combinations of parenteral/oral drugs and branded/generic drugs. These findings support the utility of S-AMU (despite the lack of direct sales) in monitoring national AMU trends in Japan. This also supports our strategy to initially publish a preliminary AMU report using S-AMU followed by a more comprehensive report using C-AMU (which includes more detailed information such as stratifications by patient age and sex) [5].

Finally, these findings also underscore a need to characterize and understand the limitations of the various data sources for AMU surveillance in order to accurately

interpret any findings, and that the appropriate surveillance method should be chosen based on the designated purpose. It is also important to adopt a "One Health" approach to controlling antimicrobial resistance, which includes monitoring AMU not only in humans, but also in animals [19]. The Japanese government's "Nippon AMR One Health Report" described the use of S-AMU to calculate the aggregate AMU in both humans and animals [20]. As sales data are used in drug utilization analyses across various epidemiological fields [21, 22], the findings of this study may be useful not only for researchers of infectious diseases, but also for other conditions.

Our study has several limitations. First, the analysis provides insight into S-AMU and C-AMU in Japan, but the characteristics and limitations of these data sources may not be directly applicable to those of other countries. For example, other countries may have a non-negligible proportion of antimicrobial sales without prescriptions [23, 24], which can lead to underestimations of C-AMU. In contrast, the inclusion of over-the-counter antimicrobial sales is not an issue in Japan due to strict regulations and easy access to prescribed antimicrobials. In this way, our findings should be evaluated in the context of Japan's healthcare system and AMU patterns. Second, information on publicly funded and self-pay patients are not available in the C-AMU. However, this may not greatly affect the longitudinal trends in C-AMU as the number of such patients

is likely to be consistent over time. Finally, we evaluated AMU using DDD values adjusted to the national population. Because longitudinal evaluations that use this metric are affected by population changes, unadjusted DDD estimates may represent a more suitable metric to evaluate AMU transitions. Nevertheless, national AMU evaluations frequently use adjusted DID as an indicator, which allows for more practical and intuitive interpretations than unadjusted values.

Conclusion

This study comparatively explored the differences in Japan's national AMU estimates from sales and claims data. While S-AMU produced higher DID estimates than C-AMU in 2013 and 2014, this trend reversed and stabilized after 2015. The differences may have been affected by the omission of direct sales information in S-AMU and the lack of electronically submitted claims in C-AMU in the earlier half of the study period. An understanding of each data source's characteristics may facilitate more accurate interpretations of national AMU estimates. These findings provide insight into the differences in Japan's S-AMU and C-AMU surveillance, and indicate that S-AMU is a viable surrogate indicator of Japan's national AMU.

Acknowledgments

None.

References

1. World Health Organization. Surveillance of Antimicrobial Use. [cited 2020 Apr 16]. Available from: http://www.who.int/medicines/areas/rational_use/AMU_Surveillance/en/
2. Pulcini C, Hulscher M. How Can We Routinely Measure Appropriateness of Antimicrobial Use in Hospitals at a National Level? JAMA Netw Open. 2019;2: e1915030. doi: 10.1001/jamanetworkopen.2019.15030
3. Pereko DD, Lubbe MS, Essack SY. Surveillance of antibiotic use in the private sector in Namibia using sales and claims data. J Infect Dev Ctries. 2016;10: 1243–1249
4. Data source overview of antimicrobial consumption. European Centre for Disease Prevention and Control. [cited 2020 Apr 17]. Available from: https://www.ecdc.europa.eu/en/antimicrobial-consumption/database/data-source-overview
5. AMR Clinical Reference Center. Surveillance. [cited 2020 Oct 30]. Available

from: http://amrcrc.ncgm.go.jp/surveillance/Surveillance_en.html

6. Muraki Y, Yagi T, Tsuji Y, Nishimura N, Tanabe M, Niwa T, et al. Japanese antimicrobial consumption surveillance: First report on oral and parenteral antimicrobial consumption in Japan (2009-2013). J Glob Antimicrob Resist. 2016;7:19–23.
7. Tsutsui A, Yahara K, Shibayama K. Trends and patterns of national antimicrobial consumption in Japan from 2004 to 2016. J Infect Chemother. 2018;24:414–21.
8. Kusama Y, Ishikane M, Tanaka C, Kimura Y, Yumura E, Hayakawa K, et al. Regional Variation of Antimicrobial Use in Japan from 2013-2016, as Estimated by the Sales Data. Jpn J Infect Dis. 2019;72:326–9.
9. Yamasaki D, Tanabe M, Muraki Y, Kato G, Ohmagari N, Yagi T. The first report of Japanese antimicrobial use measured by national database based on health insurance claims data (2011–2013): comparison with sales data, and trend analysis stratified by antimicrobial category and age group. Infection. 2018;46: 207–214.
10. Kawabe A, Muraki Y, Inose R, Kusama Y, Goto R, Ebisui A, et al. Trends of Antifungal Use Based on Sales Data in Japan from 2006 to 2015. Biol Pharm Bull. 2020;43: 1248–52.
11. Goto R, Inose R, Kusama Y, Kawabe A, Ishii S, Ebisui A, et al. Trends of the use

of anti-methicillin-resistant Staphylococcus aureus agents in Japan based on sales data from 2006 to 2015. Biol Pharm Bull (in press).

12. Matsuda S, Fujimori K. The claim database in Japan. Asian Pac J Dis Manag. 2012;6: 55–9.
13. Ministry of Health, Labour and Welfare of Japan. Situation in the prevalence of the claim submission via electronic devices (in Japanese). 2015 April [cited 2020 Apr 17]. Available from: https://www.mhlw.go.jp/stf/seisakunitsuite/bunya/0000099015.html
14. WHO Collaborating Centre for Drug Statistics Methodology. ATC/DDD Index 2019. [cited 2020 Nov 18] https://www.whocc.no/atc_ddd_index/
15. The National Statistics Center. Statistics of Japan. [cited 2020 June 1] Available from: https://www.e-stat.go.jp/en
16. Ministry of Health, Labor, and Welfare. Information of drug lists and of generic drugs (in Japanese). 2020 May 27 [cited 2020 June 1] Available from https://www.mhlw.go.jp/topics/2020/04/tp20200401-01.html.
17. Ministry of Health Labour, and Welfare of Japan. National health insurance drug price list and information of generic drugs (in Japanese). 2020 April 1 [cited 2020 Apr 17]. Available from: https://www.mhlw.go.jp/topics/2020/04/tp20200401-

01.html

18. Grüne G, Lockemann S, Kluy V, Meinhardt S. Business Process Management within Chemical and Pharmaceutical Industries. Berlin: Springer-Verlag Berlin Heidelberg; 2014.

19. White A, Hughes JM. Critical Importance of a One Health Approach to Antimicrobial Resistance. EcoHealth. 2019;16:404–9.

20. The AMR One Health Surveillance Committee. Nippon AMR One Health Report (NAOR) 2018. Tokyo: Tuberculosis and Infectious Diseases Control Division, Health Service Bureau, Ministry of Health, Labour and Welfare; 2018

21. Shibata S, Wayama Y, Tsuyuki A, Matsushita M, Chiba K, Matsuki E, et al. An Empirical Study of the Prescription Pattern of Drugs for Hematological Malignancies in Japan from 2010-2014. Biol Pharm Bull. 2017;40: 894–901.

22. Mahlich J, Kamae I, Sruamsiri R. Pharmaceutical pricing in Japan: market evidence for rheumatoid arthritis treatment. Expert Rev Pharmacoecon Outcomes Res. 2018;18: 339–348.

23. Batista AD, A Rodrigues D, Figueiras A, Zapata-Cachafeiro M, Roque F, Herdeiro MT. Antibiotic Dispensation without a Prescription Worldwide: A Systematic Review. Antibiotics (Basel). 2020;9: E786.

24. Sakeena MHF, Bennett AA, McLachlan AJ. Non-prescription sales of antimicrobial agents at community pharmacies in developing countries: a systematic review. Int J Antimicrob Agents. 2018;52: 771–82.

Response to the Editor

1. Please ensure that your manuscript meets PLOS ONE's style requirements, including those for file naming.

   Thank you for pointing this out. We have reformatted the manuscript to meet PLOS ONE's style requirements.

2. In your ethics statement in the Methods section and in the online submission form, please provide additional information about the data used in your retrospective study. Specifically, please ensure that you have discussed whether all data were fully anonymized before you accessed them and/or whether the IRB or ethics committee waived the requirement for informed consent.

   As advised, we have added the following statements to both the manuscript and the online submission system.

   Page 11 Lines 177-180
   The study did not involve any interventions in human subjects. All IQVIA data and NDB data were fully anonymized before being received by the authors. This study was approved by the institutional review board of the National Center for Global Health and Medicine (Approval Number: NCGM-G-002505-00).

3. Please describe how another researcher could obtain the data from IQVIA Japan used in this study.

   As instructed, we have included the following in the online submission form:

   Researchers should apply directly to IQVIA Japan (https://www.iqvia.com/ja-jp/locations/japan) for access to the study's antimicrobial drug sales data. The use of the criteria described in the Methods section will enable other researchers to identify the same data obtained for this study. The authors confirm that they did not have any special privileges to access the data that other researchers would not have. For the insurance claims data, the Ministry of Health, Labour and Welfare of Japan has placed strict legal restrictions on the release or sharing of these data. As a result, these data are not publicly available.

Response to Reviewers

We have made major revisions to the entire manuscript based on the reviewers' advice and suggestions. We are grateful for the opportunity to improve our work, and look forward to the reviewers' evaluation of the new structure and theories included in the manuscript. Our point-by-point responses to the comments are provided below:

Reviewer 1

This is a very interesting exploration of differences between two comprehensive, population-based methods to observe antibiotic consumption in Japan. The authors are to be commended for performing such analyses in order to understand the available data better and in order to be able to deal with limitations. It is an example for researchers in other nations to do likewise.

Thank you for your time and effort in reviewing our work, and for your positive evaluation of our manuscript. We have made major revisions in accordance with your comments and suggestions.

1. The differences found are all by all minimal, which could incite the authors to conclude that Japan has 2 pretty reliable sources to monitor antibiotic consumption. The article should clearly state that this is true for consumption in primary care (and not hospital care). It this would be incorrect it should be stated, and it should also be indicated why it is not possible to make the distinction between primary care and hospital care dispensing and reimbursement.

Thank you for the advice. S-AMU and C-AMU include both primary care data and hospital data. However, S-AMU does not contain information about where an antibiotic is used, and therefore does not allow us to distinguish between primary care and hospital care dispensing/reimbursement. As advised, we have added the following information to the Data sources subsection of the Methods.

Page 7 Lines 106-109

However, S-AMU data do not include information on the purchasing parties (i.e., names and types of medical facilities) or patients, and therefore do not allow distinctions to be made between primary care and hospital care dispensing and reimbursement.

2. Some differences were observed but not statistically evaluated. Some explanation why this did not happen might be added (too small differences, complex reversing time lines).

   As advised, we have added more statistical evaluations to our manuscript after consulting with a statistician.

   Page 14 Lines 206-212

   Effects on parenteral AMU

   For parenteral antimicrobials, both branded and generic drugs exhibited similar trends in S-AMU and C-AMU (Fig 2A). The use of generic drugs increased while the use of branded drugs decreased over the study period. Furthermore, the scatter plots of branded and generic drugs demonstrated significant correlations between S-AMU ($r = .100$, $P < .001$) and C-AMU ($r = .998$, $P < .001$) (Fig 2B). For both branded and generic drugs, S-AMU was higher than C-AMU throughout the study period.

   Page 15 Lines 223-232

   Effects on oral AMU

   The DID values of oral antimicrobials were more than 10 times higher than those of parenteral antimicrobials in both S-AMU and C-AMU. As with the parenteral antimicrobials, both branded and generic drugs in oral antimicrobials exhibited similar trends in S-AMU and C-AMU (Fig 3A). Again, the use of generic drugs increased while the use of branded drugs decreased over the study period. Throughout this period, S-AMU was higher than C-AMU for branded drugs, but C-AMU was higher than S-AMU for generic drugs. The scatter plots of branded and generic drugs demonstrated significant correlations between S-AMU ($r = .996$, $P < .001$,) and C-AMU ($r = .997$, $P < .001$) (Fig 3B).

3. The exploration of the small differences leads to hypotheses on potential sources of bias. Most of these hypotheses are plausible. For OTC sales of antibiotics in Japan, the authors states that it is illegal. That does not mean that it does not happens, even in Japan. Some indication about the actual practices in Japan, and some references to the international literature on this should be advisable. It seems plausible that this might have an influence on the observed difference in sales and claims of cheap oral antibiotics (where the remark on the legal status of OTC antibiotics was placed).

Thank you for suggesting this important point. OTC drugs are strictly regulated in Japan, and OTC antibiotics are, for all intents and purposes, not available (excluding some topical preparations). We would be hard pressed to think of any situation where a pharmacy would risk losing their retailing license and face criminal charges for illegally selling antibiotics that are easily and cheaply available with a physician's prescription. In addition, physician consultations/prescriptions are highly affordable under Japan's universal healthcare system. We have searched the international literature on the plausibility of OTC AMU in Japan, but could not find any relevant papers. In addition to the strict legislation on the sale of antibiotics, we think the main reason for this being such a non-issue is simply that the barriers to legally obtaining antibiotics are too low for illegal sales to be worth pursuing.

Although a very small number of personal trades/sales of antimicrobials through websites *may* occur, such AMU would not be reflected in sales or claims data. We have added these explanations to the limitations as follows:

Page 20 Lines 317-324

First, the analysis provides insight into S-AMU and C-AMU in Japan, but the characteristics and limitations of these data sources may not be directly applicable to those of other countries. For example, other countries may have a non-negligible proportion of antimicrobial sales without prescriptions [23, 24], which can lead to underestimations of C-AMU. In contrast, the inclusion of over-the-counter antimicrobial sales is not an issue in Japan due to strict regulations and easy access to prescribed antimicrobials. In this way, our findings should be evaluated in the context of Japan's healthcare system and AMU patterns.

We have also cited the following additional references.

23. Batista AD, A Rodrigues D, Figueiras A, Zapata-Cachafeiro M, Roque F, Herdeiro MT. Antibiotic Dispensation without a Prescription Worldwide: A Systematic Review. Antibiotics (Basel). 2020;9: E786.
24. Sakeena MHF, Bennett AA, McLachlan AJ. Non-prescription sales of antimicrobial agents at community pharmacies in developing countries: a systematic review. Int J Antimicrob Agents. 2018;52: 771–82.

Reviewer 2

The topic of the paper is of utmost importance when it comes to monitoring AMR. However, I'm under the impression that the work has been hastily prepared. The paper in my view lacks clarity and the methods does not appear sound, unless the authors are dealing with data that lack granularity. In general, thru my read, I'm left wanting more. The sources of my disappointment are as follows:

Thank you for your time and effort in reviewing our work, and we appreciate your advice and comments. The manuscript has undergone a major restructuring.

1. Lack of description of the types of variables or information that are captured by S-AMU and C-AMU

   Thank you for your useful suggestion. For greater clarity, we have added explanations of S-AMU and C-AMU as follows:

   S-AMU
   Page 7, Lines 96-109
   We estimated S-AMU using commercial data purchased from IQVIA Japan, a private data firm that curates databases of medical drug sales and distribution throughout the country. In Japan, medical drugs are generally sold by pharmaceutical companies to medical facilities via wholesalers. IQVIA Japan collects sales data from these wholesalers in order to construct databases. Although detailed information has not been published, the company states that it collects data from almost all domestic wholesalers, and that the data encompass more than 99% (monetary value) of all wholesale drug sales in Japan (this coverage was confirmed through personal communication with IQVIA Japan). Our dataset contained wholesalers' sales data of all antimicrobials, as well as information on oral/parenteral classification and branded/generic classification. However, S-AMU data do not include information on the purchasing parties (i.e., names and types of medical facilities) or patients, and therefore do not allow distinctions to be made between primary care and hospital care dispensing and reimbursement.

   C-AMU
   Page 8, Lines 112-142

We estimated C-AMU using data obtained from the National Database of Health Insurance Claims and Specific Health Checkups of Japan (NDB), which is managed by the Ministry of Health, Labour and Welfare (MHLW). The NDB contains national-level health insurance claims data for healthcare encounters covered by insurance, and these data can be used for research purposes following the submission and approval of an application to the government [12]. Our dataset included data on the dates and types of antimicrobials prescribed to patients for all insurance-covered healthcare encounters.

In Japan, all residents are required to enroll in health insurance, which entitles them to receive insurance-covered healthcare at any medical facility throughout the country. Enrollees pay monthly premiums to their insurers, and also pay a fixed proportion (10–30% depending on age and income) of the medical charges at the point of care. Healthcare providers send claims to the applicable insurers to be reimbursed for the remaining charges. Because insurance-covered care accounts for the majority of medical treatments provided in Japan, the NDB represents a near-comprehensive database of all treatments performed throughout the country.

However, the NDB does not include claims data from patients with fully publicly funded healthcare (e.g., patients with intractable diseases, atomic bomb survivors, patients on welfare, patients with tuberculosis, and patients with human immunodeficiency virus infections) and patients who personally pay for all of their medical expenses (e.g., foreign travelers and cosmetic surgery patients). The detailed numbers of these patients are not published by the government.

Furthermore, the NDB only includes data from claims that have been submitted electronically, and the lack of paper-based claims reduces its coverage of healthcare encounters. Under Japan's healthcare system, claims for medical care, dental care, and drug dispensing (by pharmacies) are handled separately. From 2011, electronically submitted claims for hospital-based medical care and drug dispensing claims have accounted for over 99.9% of total claims [13]. For clinic-based medical care, the proportions of electronically submitted claims increased from 91.0% in 2011 to 99.9% in 2017 [13]. For dental care, the proportions of electronically submitted claims have increased dramatically in recent years (31.5%, 46.4%, 55.7%, 69.5%, and 96.0% in 2011, 2012, 2013, 2014, and 2015, respectively) [13].

2. Methods look at first glance proper including potential time-series correlations but with limited granular data and its understanding, the authors are left with lots of hypotheses with some sense of triangulation of information/data

We agreed with your opinion, and have revised our study's conceptual framework and development. Previously, we had, as you pointed out, aimed to explore the biases of S-AMU and C-AMU from various aspects. But we have changed this construction: We now consider the effects of the rapid shift in paper-based claims to electronic claims in dental care as a characteristic of C-AMU, and the effects of insufficient data coverage as a characteristic of S-AMU (which is not expected to greatly affect the representativeness of the AMU situation). This simplified the study, and allowed us to focus on the correlation between S-AMU and C-AMU. The specific changes are as follows:

Page 14, Lines 209-211

Furthermore, the scatter plots of branded and generic drugs demonstrated significant correlations between S-AMU ($r = .100$, $P < .001$) and C-AMU ($r = .998$, $P < .001$).

Page 15, Lines 230-232

The scatter plots of branded and generic drugs demonstrated significant correlations between S-AMU ($r = .996$, $P < .001$,) and C-AMU ($r = .997$, $P < .001$).

3. Emphasis of the authors for the accuracy of AMU data while I believe estimating consistency of the data is much more important: I believe that the verification or validation of the consistency of the reporting from one year to another is more essential. Please recall we mean to use AMU trends as trends to be compared with AMR trends.

Thank you for the important suggestion. As advised, we have examined the longitudinal consistency of S-AMU compared to C-AMU. The specific changes are as follows:

Page 10, Lines 159-162

To evaluate the longitudinal consistency of S-AMU, we compared the annual S-AMU with C-AMU between 2013 and 2017. In addition, we compared the use of branded and generic drugs in S-AMU and C-AMU to ascertain the effect of the government's initiative to promote the shift to generic drugs.

4. Indeed, comparison between C-AMU and S-AMU is interesting (if you have access

to data, which is not the case here). Both systems could be used to monitor AMR trends as opposed to disqualifying C-AMU but is not capturing comprehensively the data.

Thank you for the suggestion. We have examined the utility of S-AMU through a comparison with C-AMU in the revised manuscript. The specific changes are as follows:

Page 18, Lines 275-276
These findings indicate that S-AMU has utility as an alternative to C-AMU for monitoring the temporal trends in national AMU.

5. Perceived some confusion re definitions between accuracy versus precision and between accuracy and biases; e.g. unclear meaning of inadequate accuracy? What does incomplete coverage mean? and moreover incomplete coverage does not mean necessary biases. E.g. incomplete data can be precise if they are representative of a situation.

Thank you for the comment. As mentioned in our response to Comment 3, we have focused the study on comparing S-AMU and C-AMU. In the revised manuscript, we consider the effects of the rapid shift in paper-based claims to electronic claims in dental care as a characteristic of C-AMU, and the effects of insufficient data coverage as a characteristic of S-AMU.

Specific comments:

6. Line 26: abstract will benefit from a reference to the reason for AMU surveillance: AMR surveillance i.e. why these AMU estimates are important?

We have clarified the importance of developing rigorous AMU surveillance systems as follows.

Page 3, Lines 26-32
Purpose
Countries require rigorous antimicrobial use (AMU) surveillance systems to monitor prescription trends and evaluate the effects of antimicrobial resistance countermeasures. AMU can be estimated using sales data (S-AMU) or insurance

claims data (C-AMU), but their differences are not well characterized. We comparatively examined Japan's national S-AMU and C-AMU estimates across a 5-year period, and explored the potential causes of their differences.

7. Line 34: unclear - as written - why the lack of paper-based claims is a limitation

   Thank you for the comment. The lack of paper-based claims potentially reduces the coverage of C-AMU. However, this may only have an effect on dental care (as reimbursements in the other types of care are predominantly managed by electronic claims). We have clarified this in the Data sources subsection of the Methods.

8. Line 36: the authors could explain why analyzing direct sales to medical facilities is important; and why medical facilities will only buy generics – how about private hospitals?

   As we have set one of the study's aims to clarify the effect of a government policy that encouraged the transition from branded drugs to generic drugs, the discussion about direct sales has become more important when compared to the previous manuscript. The description has been revised in the Discussion. In Japan, the government determines the drug prices for all administered/prescribed drugs under the national health insurance system. Therefore, patients are charged the same price for a specific drug regardless of where the drug was prescribed (public or private institutes).

9. Line 37: unclear about the importance of comparison between generic and brand drugs?

   We added the importance of comparing the trends between branded and generic drugs. This was to clarify the effect of a government policy that encouraged the transition from branded drugs to generic drugs.

   Page 6, Lines 82-85
   In addition, longitudinal consistency is a crucial aspect of a national AMU surveillance system, but antimicrobial sales may be affected by changes to healthcare policies. For example, the Japanese government has encouraged the shift from branded drugs to generic drugs from 2013 onward [10, 11].

10. Line 38: unclear significance of paper-based and electronic claims. In short, without any understanding on how it works in Japan, I'm having a hard time processing the rationality of the methods

We apologize for the inadequate explanation. We have addressed the significance of paper-based and electronic claims in the additional explanation of C-AMU, as described in our response to Comment 1.

11. Line 41 – 44: the authors share DID values and trends. How do we know that these values are statistically significantly different between each other?

Because S-AMU and C-AMU are created from different data sources, it is difficult to interpret any statistical difference. Instead, we evaluated the correlation between S-AMU and C-AMU. Please refer to our response to Comment 2.

12. Line 46: interesting that DID of brand drugs are higher than generic drugs. Reasons are not explained, and values are not shown

The DID values of each group (parenteral/oral, branded/generic, and S-AMU/C-AMU) are shown in Table 1. The higher DID for branded drugs may be due to pharmaceutical companies promoting their sale over generic drugs to healthcare providers, or a large proportion of patients and doctors who choose branded drugs based on perceptions of higher effectiveness and safety. We have added a description of the government's promotion of the transition from branded to generic drugs.

Page 6, Lines 84-85
For example, the Japanese government has encouraged the shift from branded drugs to generic drugs from 2013 onward [10, 11].

Page 18, Line 277-280
The initially high DID for branded drugs may have been due to their promotion by pharmaceutical companies and/or large proportions of patients and physicians who preferentially chose branded drugs based on perceptions of higher effectiveness and safety.

13. Line 47: what is a dispensing claim

    A dispensing claim is an insurance claim for drug dispensing by pharmacies. We have clarified the language as follows.

    Page 9, Lines 135-138
    Under Japan's healthcare system, claims for medical care, dental care, and drug dispensing (by pharmacies) are handled separately. From 2011, electronically submitted claims for hospital-based medical care and drug dispensing claims have accounted for over 99.9% of total claims [13].

14. Line 51: unclear about the relationship between "AMU is underestimated" and "lack of direct sales"; do the authors mean the data are missing or not analyzable/accessible?

    The sentence has been removed from the revised manuscript.

15. Line 51: Conclusion of abstract is weak: lots of results displayed but no indication in the conclusion to make sense of them. E.g. last sentence is a kind of catchall phrase

    We have revised the conclusion. It is difficult to meaningfully explain the results for branded/generic drugs and parenteral/oral drugs given the strict word limits of the abstract. Therefore, we have focused on the differences between S-AMU and C-AMU, and concluded that S-AMU may have utility in national AMU surveillance in Japan.

    Page 4, Lines 51-56
    Our study provides insight into the characteristics of S-AMU and C-AMU surveillance in Japan. Their differences may have been influenced by the omission of direct sales information in S-AMU and the lack of electronically submitted claims for dental care in C-AMU. However, these factors did not appear to differentially affect the temporal trends. The differences stabilized after 2015, suggesting that S-AMU is a viable surrogate indicator of Japan's national AMU.

16. Line 73: the authors argue that previous data did not account for time-series

correlations. However, it appears the present study does not show any time-series correlation analysis either, does it?

We have added an analysis of the correlation of S-AMU and C-AMU according to year. Please refer to our response to Comment 2.

17. Line 84: this paragraph on data sources should be expanded to insert much more information on Japan's healthcare systems, detailed description of what is available in terms of variables and time periods of C-AMU and S-AMU. E.g. Private vs public hospitals; vet antibiotics versus antibiotics for humans; only one private company supplying Japan?

    We have added more detailed explanations of S-AMU and C-AMU in our response to Comment 1. Furthermore, as advised, we have addressed veterinary AMU in the Discussion with newly cited reports.

    Page 20, Lines 310-314
    It is also important to adopt a "One Health" approach to controlling antimicrobial resistance, which includes monitoring AMU not only in humans, but also in animals [19]. The Japanese government's "Nippon AMR One Health Report" described the use of S-AMU to calculate the aggregate AMU in both humans and animals [20].

18. Line 105: meaning of inadequate accuracy? Is it important that the data is inaccurate if on can demonstrate that the data is precise and more importantly consistent overtime? If the authors claim inaccuracy – then what is the extent of the inaccuracy?

    We have made major revisions to the manuscript, and the sentences have been deleted.

19. Line 106: just so that I'm clear: S-AMU data pertain to 99% of total sales but direct sales data to medical facilities are missing or not broken down or categorized as such in the dataset?

    We apologize for the inadequate explanation. S-AMU data included 99% of wholesale data.

20. Line 117: would be interesting to mention how different these data are different from

that of Europe in terms of available variables

We searched for lists of available variables of S-AMU in European countries, and contacted IQVIA about these variables. Unfortunately, we could not obtain any lists that could support such a comparison.

21. Line 120: what is the extent of the AMU among tourists and for cosmetic procedures? Does it matter if we consider the inaccuracy is consistent over time? Recall that trends matter more for AMU and AMR surveillance purposes

    We agree with your comments. We have included a study aim to examine the longitudinal consistency between S-AMU and C-AMU, and addressed this lack of data from C-AMU in the limitations.

    Page 20, Lines 324-327
    Second, information on publicly funded and self-pay patients are not available in the C-AMU. However, this may not greatly affect the longitudinal trends in C-AMU as the number of such patients is likely to be consistent over time.

22. Line 127: what kind of data is shared by IQVIA? Are there any limitations in relation to data sharing? Please detail what is shared by the company and what is available?

    We have added a more detailed explanation of the IQVIA data. The purchased data only contained sales of drugs from wholesalers to buyers (mainly medical facilities). IQVIA has restrictions prohibiting the public sharing of the data. However, researchers are free to purchase similar data directly from IQVIA.

23. Line 153: again comparison between the 2 AMU data could be pertinent if these DID values are statistically significant or not?

    Thank you for your advice. We have examined the correlations between the 2 AMU data types as detailed in our response to Comment 2.

24. Line 169: IQVIA accounts for 99% of the sales – is this for antimicrobials or all drugs? if data is made available, wouldn't it be interesting to compare sales of antibiotics with that of other drugs, trends wise? Do you mean IQVIA has the

monopoly of sales?

IQVIA accounts for 99% of all wholesale drug sales. Unfortunately, we had only purchased data regarding antimicrobials. While other vendors are free to collect and offer similar data, there are currently none that do so.

25. Line 170: Cant we work out some collaboration with the company for public health purposes to have access to more granular data?

    Thank you for your suggestion. IQVIA is unable to provide more granular data due to confidentiality agreements with the wholesalers.

26. Line 175-176: the main result in my view is the consistency of differences over many of these factors (parental vs per os, generic/branded). Shouldn't the authors emphasize this more in the results?

    Thank you for your suggestions. We have revised our study framework, and emphasize the consistency of the differences between generic vs branded drugs in both oral and parenteral drugs. Please refer to our response to Comment 2.

27. Line 181 – 182: 2 sentences that say the same thing
    We have revised the sentence as follows:

    Page 15, Lines 228-230
    Throughout this period, S-AMU was higher than C-AMU for branded drugs, but C-AMU was higher than S-AMU for generic drugs.

28. Line 185: figure 2 seems to show interesting trends over time but no analysis?

    We added Pearson's correlation test to these results. Please refer to our response to Comment 2.

29. Line 198: only one seems to realize that paper-based claims are not submitted into the system.

    We agree with your opinion. We have revised the discussion of paper-based claims

such that it is now considered a characteristic of C-AMU.

30. Line 220 -221: not sure I understand what sort of impact the authors were referring to

    This sentence was deleted as part of the major revisions to the manuscript.

31. Line 222: difficult to understand this with limited background on how antimicrobials are supplied and distributed

    We have added the following explanation about this statement.

    Page 18, Lines 285-289
    In general, pharmaceutical companies sell their products to medical facilities through wholesalers to reduce the burden of sales. However, some companies may sell their products directly to medical facilities to increase their profit margins that would otherwise be lost to the wholesalers.

32. Line 230: the paper becomes much more interesting if there is some attempt to estimate the extent of the biases. Unfortunately, we don't have this in this paper

    Although we agree with this point, we were unable to quantify the extent of these biases with our data. Nevertheless, the study has shifted from speculating about these possible biases, and focused on the differences between C-AMU and S-AMU.

33. Line 245: I disagree with authors; C-AMU is valuable if consistent overtime

    We have changed the discussion according to your advice, and regarded C-AMU as the standard method for evaluating the national AMU. Please refer to our response to Comment 4.

34. Line 247: this information could be shared earlier in a potential section on detailed description of AMU data

    Thank you for the comment. However, the need for human-animal aggregate AMU is, although important, not central to our study aims. We have therefore kept this issue

in the Discussion.

35. Line 260: why not doing the analysis of unadjusted DDD in this paper as this paper contains limited information and results

The reason for using population-adjusted DDD is written in the limitations. Adjusted DID is a more frequently used metric of national AMU than unadjusted DDD, with major international organizations (e.g., WHO and ECDC) using this unit to evaluate the AMU of countries. If readers would like to evaluate unadjusted DDD, they can easily obtain the figures by multiplying DID with the population of Japan, as described in Reference #15 (Statistics of Japan).

Page 21, Lines 327-332
Finally, we evaluated AMU using DDD values adjusted to the national population. Because longitudinal evaluations that use this metric are affected by population changes, unadjusted DDD estimates may represent a more suitable metric to evaluate AMU transitions. Nevertheless, national AMU evaluations frequently use adjusted DID as an indicator, which allows for more practical and intuitive interpretations than unadjusted values.
