# Supplementary material for: Characteristics and limitations of national antimicrobial surveillance according to sales and claims data
Source: PLoS One. 2021 May 11;16(5):e0251299. doi: 10.1371/journal.pone.0251299 (PMC8112693; doi:10.1371/journal.pone.0251299)
Supplement: Attachment — Submitted filename: PONE-D-20-21601_R2_Response to editor and reviewers.docx [file pone.0251299.s003.docx]

**Response to editor’s comments**

Thank you for reviewing our manuscript and providing helpful comments. We have thoroughly reconsidered the fundamental structure of the manuscript based on the editor and reviewer comments. In particular, we have clarified the objective of the study, namely, elucidating the characteristics and limitations of S-AMU and C-AMU. To evaluate these objectives, we created monthly S-AMU and C-AMU trends and performed corresponding statistical analyses.

The major revisions are as follows:

- We changed the observation time-scale from yearly to monthly.
- We evaluated the S-AMU/C-AMU ratio (SCR) to assess trends in the relationship between S-AMU and C-AMU.
- We removed the comparison of penicillin and fluoroquinolone because it was ambiguous.
- We changed the method of analysis from correlation analysis to linear regression for the comparison of S-AMU and C-AMU.
- We changed the title, abstract, and Discussion section, accordingly.

Our point-by-point answers are as follows.

1. The new to state more clearly what is the central research question of the paper, and present results as main finding, and then broken down into more detailed conclusions. If the main goal of the paper to investigate whether S-AMU data can be used as a surveillance tool as a substitute to C-AMU? Then needs to be stated clearly. Right now the abstract and introduction go back and forth between saying that the paper will look for differences between the two sources, and that the paper will look for similarities (correlation). These seem apparently contradicting unless clearly stated as parts of the overarching goal. Which is not to just compare the two, but specifically to see if S-AMU can be considered as good as C-AMU for the purpose of surveilling trends in usage.

Thank you for pointing this out. We completely agree that the stated objectives were inconsistent. Accordingly, we have reconsidered the fundamental structure of the manuscript and revised nearly all of it from the beginning.

2. The statistics needs much improvement. All statements made should be defended by statistical results (avoid statements like "consistently higher", and instead give statistical results and confidence). Regarding 2), I question in particular why data were aggregated yearly, rather than for instance monthly. Doing correlation and trend analysis with 5 data points may never give the confidence you need to backup the conclusions you are trying to make. Please pay particular attention to the reviewer's comments on the figures (there seems to be a contradiction between what the text says about the figures 2B and 3B, and what they actually show). Please review the wording on the sentence in lines 64-66.

In accordance with your comment, we have changed the basic time-scale of the analysis from yearly to monthly. We have been careful to describe the theory in statistical terms and have revised nearly the entire manuscript, including the title, abstract, and Figures. I would be very grateful if the editors could read and evaluate our revised manuscript.

Reviewer #1: The authors have responded well to the two reviewers' comments. There are no further recommendations to be made.

We sincerely appreciate your kind review.

Reviewer #2:

Thank you for reviewing our manuscript and providing helpful comments. We have thoroughly reconsidered the fundamental structure of the manuscript based on the editor and reviewer comments. In particular, we have clarified the objective of the study, namely, elucidating the characteristics and limitations of S-AMU and C-AMU. To evaluate these objectives, we created monthly S-AMU and C-AMU trends and performed corresponding statistical analyses.

The major revisions are as follows:

- We changed the observation time-scale from yearly to monthly.
- We evaluated the S-AMU/C-AMU ratio (SCR) to assess trends in the relationship between S-AMU and C-AMU.
- We removed the comparison of penicillin and fluoroquinolone because it was ambiguous.
- We changed the method of analysis from correlation analysis to linear regression for the comparison of S-AMU and C-AMU.
- We changed the title, abstract, and Discussion section, accordingly.

I would like to congratulate the authors for carefully addressing the reviewer’s comments. the ms is now much clearer and I do understand the approach and the findings better. However, in my view I would encourage to have a second look of the results and discussion sections. Results are in my opinion are not well presented with limited used of statistical tools e.g. trends and not values should be the focus; Discussion is confusing and not well presented. e.g. the authors should start with the major findings, the major recommendations and break down into secondary findings. Please find attached my specific comments.

Thank you for your meticulous suggestions. In accordance with your comments, we have reconsidered the fundamental structure of the manuscript and revised nearly all of it from the beginning. Our point-by-point responses are as follows.

**Abstract**

1. Line 46 what is the overall result? to me this is not the main finding. you can break down later.
   We have presented our findings numerically and statistics-based terms to the extent possible in the revised manuscript. Please let us know if you have further concerns or suggestions.
2. Line 46 what does it mean - difference is correlated or trends is correlated?

We have clarified the statistical methods in both the abstract and the main text.

3. Line 47 where are the p values for these comparisons?

We have presented our findings numerically and statistics-based terms to the extent possible in the revised manuscript. Please let us know if you have further concerns or suggestions.

4. Line 51 overall results? It should be presented first.

We have revised the conclusion accordingly. Specifically, we now present the overall results descriptively, rather than numerically.

5. Line 55 do you mean that C-AMU is not good? why?

We did not intend to imply that C-AMU is not good. Accordingly, we have revised the description for clarification. Both types of AMU are useful depending on the situation.

The revised abstract is as follows.

**Abstract**

Purpose

Antimicrobial use (AMU) is estimated at the national level by using sales data (S-AMU) or insurance claims data (C-AMU). However, these data might be biased by generic drugs that are not sold through wholesalers (direct sales) or recorded in sales databases, or by claims that are not submitted electronically or stored in claims databases. We evaluated these effects by comparing S-AMU and C-AMU to ascertain the characteristics and limitations of each kind of data. We also evaluated the interchangeability of these data by assessing their relationship.

Methods

We calculated monthly defined daily doses per 1,000 inhabitants per day (DID) using sales and claims data from 2013 to 2017. To assess the effects of non-electronic claim submissions on C-AMU, we evaluated trends in the S-AMU/C-AMU ratio (SCR). To assess the effects of direct sales of S-AMU, we divided AMU into generic and branded drugs and evaluated each SCR in terms of oral versus parenteral drugs. To assess the relationship between S-AMU and C-AMU, we created a linear regression and evaluated its coefficient.

Results

Median annual SCRs from 2013 to 2017 were 1.046, 0.993, 0.980, 0.987, and 0.967, respectively. SCRs dropped from 2013 to 2015, and then stabilized. Differences in SCRs between branded and generic drugs were significant for oral drugs (0.820 vs 1.079) but not parenteral drugs (1.200 vs 1.165), suggesting that direct sales of generic drugs were omitted in S-AMU. Coefficients of DID between S-AMU and C-AMU were high (generic, 0.90; branded, 0.84) in oral drugs but relatively low (generic, 0.32; branded, 0.52) in parenteral drugs.

Conclusions

The omission of direct sales information and non-electronically submitted claims have influenced S-AMU and C-AMU information, respectively. However, these data were well-correlated, and it is considered that both kinds of data are useful depending on the situation.

**Instruction**

6. Line 80 does this study have the same period as the study of the present ms?

We have added the study periods of the reference as follows.

Line 77-81
For C-AMU, the scope of medical care covered by health insurance is a key factor in the validity of the dataset. In Japan, nearly all medical care is covered by the national health insurance system, with a few exceptions such as cosmetic surgery and travel medicine (the number of items covered has not changed over time, according to the study conducted from 2011 to 2013 [8]).

**Methods**

7. Line 108 no distinction either regarding geographic locations of those facilities?

Although the geographic locations are registered in the database, we cannot use that information because of IQVIA Japan’s rules concerning data provision.

8. Line 146 please remind what J01 is about

We have added an explanation of J01 as follows.

Line 152-153

Antimicrobials are identified using the Anatomical Therapeutic Chemical (ATC) classification J01 (anti-infectives for systemic use)

9. Line 149 what is a maintenance dose

We have added an explanation of maintenance dose as follows.

Line 158 (i.e., not loading dose)

**Results**

10. Line 191 I don’t see a P correlation coef value and its p value?

We have added the following statistical information to this part.

Lines 211-218

Differences in SCRs over 5 years were significant (Kruskal-Wallis test, P = 0.002). Furthermore, there were significant differences in SCRs among the years 2013, 2014, and 2015, even after Bonferroni correction (Kruskal-Wallis test, P = 0.002). Meanwhile, significant differences were not observed among the years 2015, 2016, and 2017. (Kruskal-Wallis test, P = 0.926). Panel A of Figure 1 shows that the SCR for most months was less than 1 from 2015, meaning that C-AMU was higher than S-AMU.

11. Line 203 where are the comparison values and p values?

The tables now show the DID values (for replication studies), and the *P* values are now presented in the footnote of the figures.

Lines 227-231, Figure 1

Panel A shows the temporal trends in SCRs. Panel B shows the annual SCRs. SCR was highest in 2013, then decreased and stabilized after 2015. Difference in SCRs over 5 years were significant (P = 0.002). SCRs were significantly different among the years 2013, 2014, and 2015 (P = 0.002), but not among the years 2015, 2016, and 2017. (P = 0.926).

12. Line 211 I don’t understand these values - what are you comparing? and these values don’t appear in fig 2 either

We assessed the coefficient of the linear regression between S-AMU and C-AMU. The results of the statistical analysis are shown in the footnote of Figure 3.

Line 272, Figure 3

Coefficients (P value) and adjusted R squared values for oral drugs were 0.90 (P < 0.001), 0.92 in generic drugs, and 0.84 (P < 0.001), 0.91 in branded drugs, and for parenteral drugs, were 0.32 (P < 0.001), 0.43 in generic drugs, and 0.52 (P < 0.001), 0.40 in branded drugs.

13. Line 212 what is higher?

We have removed this sentence.

14. Line 218 sorry I dont understand the significance of it; if the authors want to correlate, it should be by year... using another type of graph

We consider that this issue has been resolved by the use of monthly plots.

15. Line 231 I understand now what these 2 values of coefficients refer to but not correctly presented - please revise not shown in figure 3 as well - they should

We have assessed the coefficients of the linear regression between S-AMU and C-AMU, as described in our response to comment #12.

16. Line 244 please present these data in trends - very difficult to read adn compare trends - presenting ranges of values are to me secondary!

Thank you for this suggestion. We rewrote the manuscript in statistical terms to the extent possible. Accordingly, we judged that this figure (trends in DIDs of penicillin and fluoroquinolone) was no longer necessary and so it has been removed from the manuscript.

**Discussion**

17. Line 260 In my view, the authors should make an effort to present the discussion in a more classic way:

- start with the main finding and consequences (I had to wait for the conclusion to understand the main recommendations)

- other findings that are less secondary and limitations could come later

We have revised the Discussion section for flow and clarity.

18. Line 263 again, to me, this is not about values but trends:

- trends differ during 2013-2015

- trends are the same over the next 3 years

would these trends be similar, both systems can be used interchangeably to monitor the impact of AMU over AMR trends... correct? For practicality reasons, we recommend C AMU as it is made available .... correct?

We now present these two points in statistical terms and have revised the relevant text for clarity.

19. Line 264 this sentence is not clear - please revise

The description has been revised along with the changes to the rest of the manuscript. I believe this part is now clearer than before.

20. Line 276 don’t understand - why not use both? do we need to choose?

Yes, we should use both types of data depending on the situation. We have revised this description as follows.

Lines 309-311

These findings indicate that we can use both S-AMU and C-AMU to monitor national AMU. Therefore, when these characteristics and limitations are sufficiently understood, we can select the surveillance method according to the target purpose.

21. Line 284 ?? posit?

We have deleted the word “posit”.

22. Line 300 this is the main finding

Thank you for noting this. We now present this as the main finding of the study.
